# Transcriptomics of temperature-sensitive *R* gene-mediated resistance identifies a *WAKL10* protein interaction network

**DOI:** 10.1038/s41598-024-53643-7

**Published:** 2024-02-29

**Authors:** Katherine Noel, Ivan R. Wolf, David Hughes, Guilherme T. Valente, Aiming Qi, Yong-Ju Huang, Bruce D. L. Fitt, Henrik U. Stotz

**Affiliations:** 1 Centre for Agriculture, Food and Environmental Management, University of Hertfordshire, Hatfield, AL10 9AB UK; 2 Department of Biological Sciences, University of North Carolina, Charlotte, NC 28223 USA; 3 Intelligent Data Ecosystems, Rothamsted Research, Harpenden, AL5 2JQ UK; 4 School of Medicine, São Paulo State University – UNESP, Botocatu, SP 18618687 Brazil; 5 Present Address: LS Plant Breeding, North Barn, Manor Farm, Milton Road, Cambridge, CB24 9NG UK

**Keywords:** Plant sciences, Plant immunity, Effectors in plant pathology, Environmental sciences, Environmental impact

## Abstract

Understanding temperature-sensitivity of *R* gene-mediated resistance against apoplastic pathogens is important for sustainable food production in the face of global warming. Here, we show that resistance of *Brassica napus* cotyledons against *Leptosphaeria maculans* was temperature-sensitive in introgression line Topas-*Rlm7* but temperature-resilient in Topas-*Rlm4*. A set of 1,646 host genes was differentially expressed in Topas-*Rlm4* and Topas-*Rlm7* in response to temperature. Amongst these were three *WAKL10* genes, including BnaA07g20220D, representing the temperature-sensitive *Rlm7-1* allele and *Rlm4*. Network analysis identified a *WAKL10* protein interaction cluster specifically for Topas-*Rlm7* at 25 °C. Diffusion analysis of the Topas-*Rlm4* network identified *WRKY22* as a putative regulatory target of the ESCRT-III complex-associated protein *VPS60.1*, which belongs to the *WAKL10* protein interaction community. Combined enrichment analysis of gene ontology terms considering gene expression and network data linked vesicle-mediated transport to defence. Thus, dysregulation of effector-triggered defence in Topas-*Rlm7* disrupts vesicle-associated resistance against the apoplastic pathogen *L. maculans*.

## Introduction

Climate change not only affects the stability and resilience of crop food systems, threatening food security, but also increases severity of crop disease outbreaks, causing losses in crop yield and quality^1^. Warmer climates have contributed to the spread of diseases, including Pierce’s disease of grapevines, citrus and olive trees caused by vector-borne bacterium *Xylella fastidiosa* and stem rust of wheat caused by *Puccinia graminis* f. sp. *tritici* (*Pgt*)^2,3^. Climate change scenarios suggest that severity of phoma stem canker of oilseed rape (*Brassica napus*) caused by the apoplastic dothidiomycete *Leptosphaeria maculans* will increase in the UK under future warming^4,5^.

Plant defence against infectious diseases consists firstly of an innate immune response through recognition of pathogen-associated molecular patterns (PAMP) by pattern recognition receptors^6^. Adapted pathogens have evolved effectors to overcome PAMP-triggered immunity (PTI). Conversely, plants have evolved *R* gene-mediated resistance to activate effector-triggered immunity (ETI) or effector-triggered defence (ETD) in the case of *B. napus* versus *L. maculans* to defeat adapted pathogens^5,7^. Interaction between *Arabidopsis thaliana* and *Pseudomonas syringae* has higher (23–32 °C) and lower (10–23 °C) temperature optima for PTI and ETI, respectively^8^. While some *R* genes confer temperature-resilient resistance, like *Sr21* operating against Ug99 race of *Pgt*^9^, there are many examples of temperature-sensitive *R* genes, including the *N* gene of tobacco^10^. *R* gene-mediated resistance of *B. napus* against *L. maculans* operates at ≤ 20 °C, but not always at ≥ 25 °C^11,12^. Information about temperature-sensitive resistance against *L. maculans* is limited. Few of 19 identified *R* genes have been tested for their temperature response^11–13^; *LepR3* and *Rlm2* encode receptor-like proteins (RLPs), but *Rlm9*, *Rlm4* and *Rlm7* encode wall-associated kinase-like 10 (*WAKL10*) genes^14–16^. Whereas *LepR3* and *Rlm2* recognise corresponding *AvrLm1* and *AvrLm2* effector genes of *L. maculans*, respectively, *Rlm4* and *Rlm7* recognise the common effector *AvrLm4-7*^17^.

*AtWAKL22* was perhaps the first gene of this family implicated in disease resistance^18^. The *Stb6* gene of wheat, *TaWAKL4*, confers resistance against the apoplastic pathogen *Zymoseptoria tritici*^19^. This pathogen has a similar infection strategy to *L. maculans*, entering the host through stomatal pores and colonising the mesophyll layer without penetrating host cells^20^. The *Rrs1* resistance locus of barley against the subcuticular pathogen *Rhynchosporium commune* features three transcripts with wall-associated receptor kinase domains^21^. The quantitative resistance locus *Htn1* of maize against appressorium-forming pathogen *Exserohilum turcicum* involves *ZmWAK-RLK1*^22^. *Snn1* of wheat is a *TaWAK* gene that confers susceptibility to the necrotrophic pathogen *Parastagonospora nodorum* producing the *SnTox1* protein^23^. *WAKL* genes therefore function in a variety of pathogen contexts and relate to *R* gene-mediated resistance against apoplastic pathogens.

Molecular mechanisms of *WAKL* function relate to its protein domains. Whereas the extracellular domain includes a galacturonan-binding domain (PF13947), the intracellular portion comprises a Ser/Thr protein kinase domain with a guanylyl cyclase (GC) centre^16,22^. Evidence for GC and protein kinase activities was obtained by in vitro biochemical analysis of *AtWAKL10*^24^. Responsiveness of the extracellular domain of *AtWAK1* to oligogalacturonides (OGs) and triggering of the OG response pathway by the intracellular *AtWAK1* domain were shown using domain swaps^25^. Such moonlighting proteins with multiple activities generate signalling niches and are involved in mechanical stress responses^26,27^.

Changes in pathogen and host gene expression during interactions between *B. napus* and *L. maculans* have been documented^28,29^. *Rlm2*-mediated resistance against *L. maculans* involved RLP-dependent up-regulation of genes participating in hormone responses, Ca^2^^+^ and mitogen-activated protein kinase (MPK) signalling^30^. Salicylic acid (SA)-dependent defence response pathways were consistently activated during *R* gene-mediated resistance with coincident induction of the pathogenesis-related *PR1* gene^30–32^. *PR1* accumulates locally after pathogen infection^29^, and proteolytic processing of PR1 releases the C-terminal CAP-derived peptide 1 to activate defence signalling^33,34^. *WAKL* genes are *L. maculans*-responsive^29,32^. *WRKY* transcription factors regulate *AtWAKL10* expression^35^. As part of the PTI response, *OsWAK* genes are responsive to chitin in rice^36^.

Adaptation to climate change is an increasingly important aspect of plant breeding, and temperature-resilient resistance against pathogens remains a crucial goal. However, little is known about pathways involved in temperature-sensitive resistance in crops, although temperature-dependent trade-offs between plant defence and growth are better known in *A. thaliana*^37,38^. This paper aims to better understand mechanisms of temperature-dependent resistance against crop pathogens by focussing on the *L. maculans*/*B. napus* pathosystem. Doubled-haploid (DH) Topas introgression lines (ILs) carrying *Rlm7* or *Rlm4* were used to study temperature-sensitive resistance against *L. maculans* carrying the corresponding *AvrLm4-7* effector. Transcriptomics was used to identify differentially expressed genes (DEGs) that responded to temperature in an IL-dependent manner. Protein interaction and regulatory network analysis provided new insights into temperature sensitivity of ETD against an economically important global crop pathogen.

## Results

### Differences between Topas-*Rlm4* and Topas-*Rlm7* in temperature-sensitive resistance against *L. maculans*

**Figure 1:**
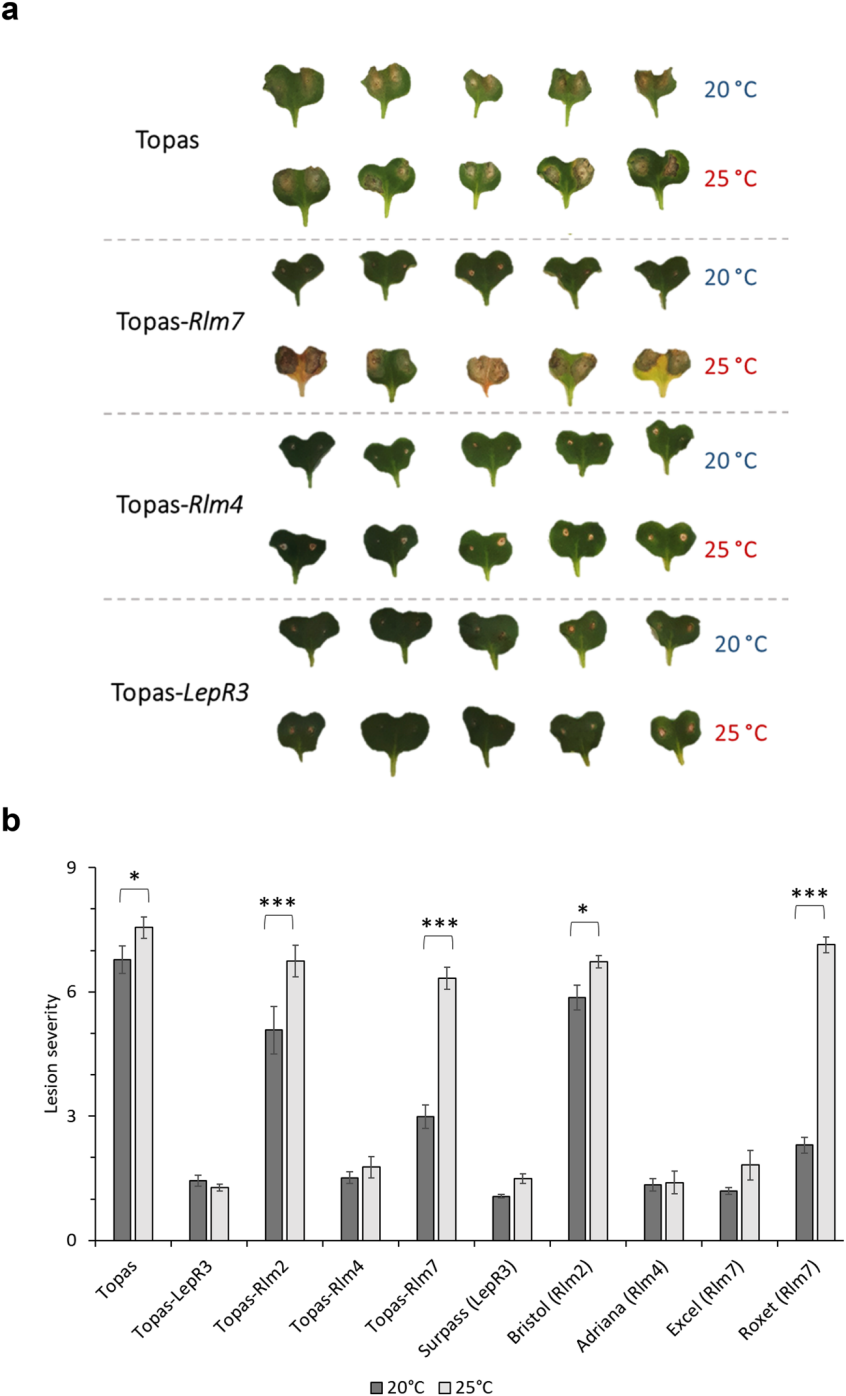
Symptoms and lesion severity of different *Brassica napus* lines and cultivars at 20 °C and 25 °C. (**a**) Cotyledons of the susceptible doubled-haploid background Topas and its single *R* gene introgression lines (Topas-*Rlm7*, Topas-*Rlm4* or Topas-*LepR3*) were point-inoculated with 10 µl of 10^7^ spores ml^−1^ conidial suspensions of *Leptosphaeria maculans* isolate JN3 (*AvrLm1-4-5-6-7-8*). Cotyledons were photographed at 12 days post-inoculation (dpi). (**b**) Average lesion severity, 0 (resistant) to 9 (susceptible) scale, assessed on cotyledons of Topas introgression lines containing *LepR3*, *Rlm2*, *Rlm4* or *Rlm7*, and a differential set of cultivars containing each of these *R* genes, after point-inoculation with 10 µl of 10^7^ ml^−1^ conidial suspension of isolate JN3 at 13 dpi. Four sites were assessed per plant. Eight biological replicates were included for each of the introgression lines and six biological replicates were included for each the differential set of cultivars for which each assay was done twice. Bars represent mean lesion scores and error bars show standard errors of the mean (*P < 0.05, ***P < 0.001).

To determine temperature-sensitivity of *R* gene-mediated resistance, cotyledons were inoculated at 20 °C or 25 °C using a set of *B. napus* Topas ILs and differential cultivars. Topas DH16516 (referred to as Topas), used as control, was susceptible to *L. maculans* at 20 °C and 25 °C (Fig. 1). Topas-*Rlm7* was resistant against an avirulent *L. maculans* isolate at 20 °C but susceptible at 25 °C with large lesions forming, in contrast to Topas-*Rlm4* and Topas-*LepR3* that were resistant at both temperatures.

Quantitative differences in *R* gene-mediated resistance of Topas ILs (*LepR3*, *Rlm2*, *Rlm4* and *Rlm7*) and a differential set of cultivars were assessed at 20 °C and 25 °C using *L. maculans* isolates JN3 (*AvrLm1-4-5-6-7-8*) and 99–79 (*AvrLm2-4-7*) (Fig. 1b, Supplementary Fig. 1). *Rlm4* was temperature-resilient in both Topas-*Rlm4* and cultivar (cv.) Adriana (containing *Rlm4*), independent of the isolate challenge. *LepR3* was also temperature-resilient in Topas-*LepR3* and cv. Surpass (containing *LepR3*) when inoculated with JN3; isolate 99–79, not containing *AvrLm1*, triggered a susceptible response in *LepR3* genotypes (Supplementary Fig. 1). Cultivar Roxet (containing *Rlm7*) was temperature-sensitive when inoculated with JN3. Thus, *Rlm7* was temperature-sensitive in the Topas-*Rlm7* and in cv. Roxet, from which the *Rlm7* was introgressed^39^ but temperature-resilient in cv. Excel.

To determine whether differences in phenotype were a function of pathogen growth rate, axenic growth of *L. maculans* was assessed at temperatures 20 °C and 25 °C. Both isolates showed no significant differences in growth rate between these temperatures (Supplementary Fig. 2).

### The defence response against *L. maculans* involves protein phosphorylation

**Figure 2:**
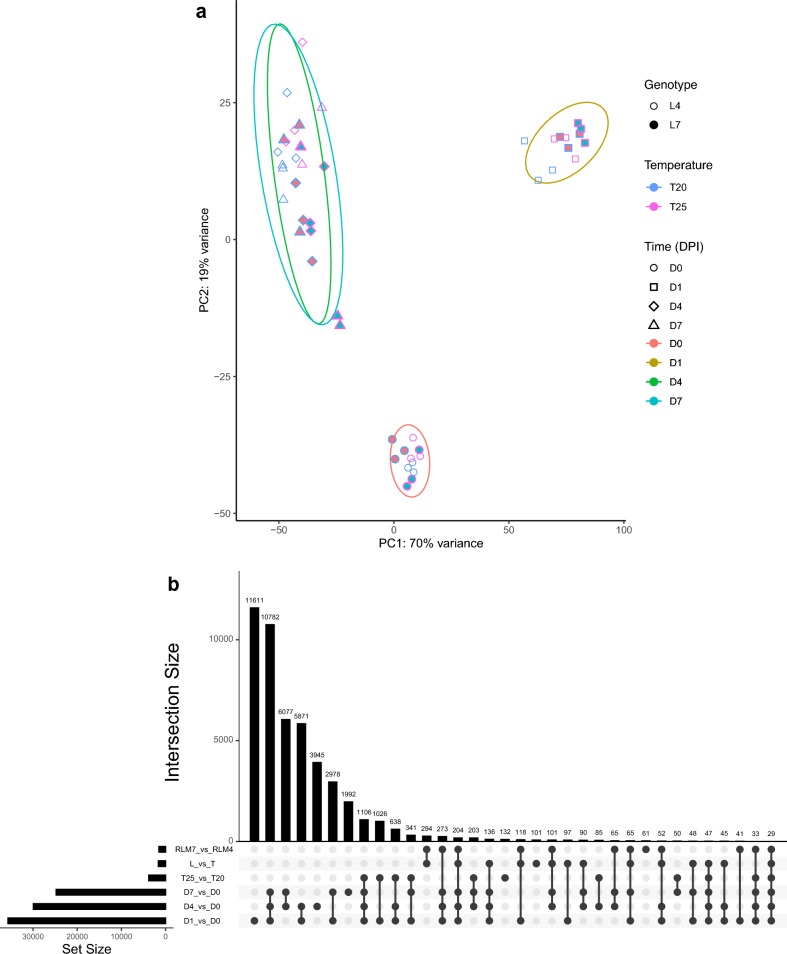
RNA-seq analysis of *Brassica napus* introgression lines Topas-*Rlm4* or Topas-*Rlm7* at 0-, 1-, 4- or 7-days post-inoculation (dpi) with *Leptosphaeria maculans* isolate JN3 (*AvrLm1-4-5-6-7-8*) at 20 °C or 25 °C. (**a**) Principal component (PC) analysis of RNA-seq samples. Host transcriptome data were separated by line Topas-*Rlm4* (L4, open symbols) and Topas-*Rlm7* (L7, filled symbols), temperature 20 °C and 25 °C (blue versus red outlines) and time 0, 1, 4 and 7 dpi (different shapes). Ellipses outline areas of 95% confidence for the different time-points. (**b**) Intersection between differentially expressed genes (DEGs) in *B. napus* after inoculation with *L. maculans*. UpSet plot showing temporal differences in numbers of DEGs at 1 (D1 vs. D0), 4 (D4 vs. D0) and 7 (D7 vs. D0) dpi. The effect of temperature on DEGs at 20 °C versus 25 °C (T25 vs T20) is shown, as is the effect of introgression line (RLM7 vs RLM4); the line x temperature (L vs T) interaction effect relates to DEGs in Topas-*Rlm4* versus Topas-*Rlm7* at these two temperatures. DEGs were defined using *P*_adj_ < 0.01. Interaction sizes less than 29 were removed to improve plot resolution.

To better understand molecular mechanisms of *R* gene-mediated temperature-sensitive resistance, an RNA sequencing (RNA-seq) study was done. Topas-*Rlm7* and Topas-*Rlm4* were compared at 20 °C and 25 °C during early stages of infection. Principal component analysis (PCA) using *B. napus* (Fig. 2a) and *L. maculans* datasets (Supplementary Fig. 3) separated the early infection phase at 1-day post-inoculation (dpi) from later time-points at 4 and 7 dpi as well as the control at 0 dpi. Principal component 2 (PC2) differentiated between infected and control samples, whereas PC1 strongly differentiated stages of infection, i.e. 1 dpi versus 4 and 7 dpi. Genotypic differences between Topas-*Rlm7* and Topas-*Rlm4* were evident at 4 dpi (Supplementary Fig. 4). PCA of the fungal transcriptome reflected the effect of time observed for host gene expression, although PC1 separated infected and control samples whereas the stages of colonisation were separated by PC2 (Supplementary Fig. 3).

Numbers of DEGs were determined by the main effects of time, temperature and line and line-by-temperature interaction (Table 1). Numbers of up-regulated and down-regulated host (Fig. 2b) and pathogen DEGs continuously decreased from 1 dpi to 4 and 7 dpi. The proportion of DEGs influenced by temperature was greater in the host than the pathogen (Table 1). Pathogen DEGs were neither influenced by line nor by line-by-temperature interaction and therefore could be excluded as a factor influencing temperature-sensitive resistance in Topas-*Rlm7*. Although one third of the DEGs expressed at 1 dpi were stage-specific, only 13% and 8% were stage-specific at 4 and 7 dpi, respectively (Fig. 2b). DEGs shared between 4 and 7 dpi had more in common than DEGs shared between 1 and 4 dpi or between 1 and 7 dpi, in agreement with the PCA plot (Fig. 2a). The majority of the temperature-sensitive DEGs was represented at the different time points of infection. The largest fraction of DEGs in the line-by-temperature category (18%) was in common with the line category, in contrast to the largest interaction size between temperature and line-by-temperature categories (3%).

**Table 1:** Numbers of differentially expressed genes (DEGs) in *Brassica napus* introgression lines Topas-*Rlm4* and Topas-*Rlm7* after inoculation with *Leptosphaeria maculans* for different lengths of time at different temperatures.

	*B. napus*		*L. maculans*	
	Up-regulated	Down-regulated	Up-regulated	Down-regulated
Day 1 vs Day 0^1^	17,487	18,195	6749	32
Day 4 vs Day 0	15,762	14,160	6206	23
Day 7 vs Day 0	13,228	11,538	6168	22
Temperature (25 °C vs 20 °C)	1743	2086	40	8
Line (*Rlm7* vs *Rlm4*)	792	726	0	0
Line x Temperature	733	913	0	0

^1^Five main effects and an interaction term are shown using normalised expression with the vsd function of the DESeq2 package; P_adj_ < 0.01.

**Figure 3:**
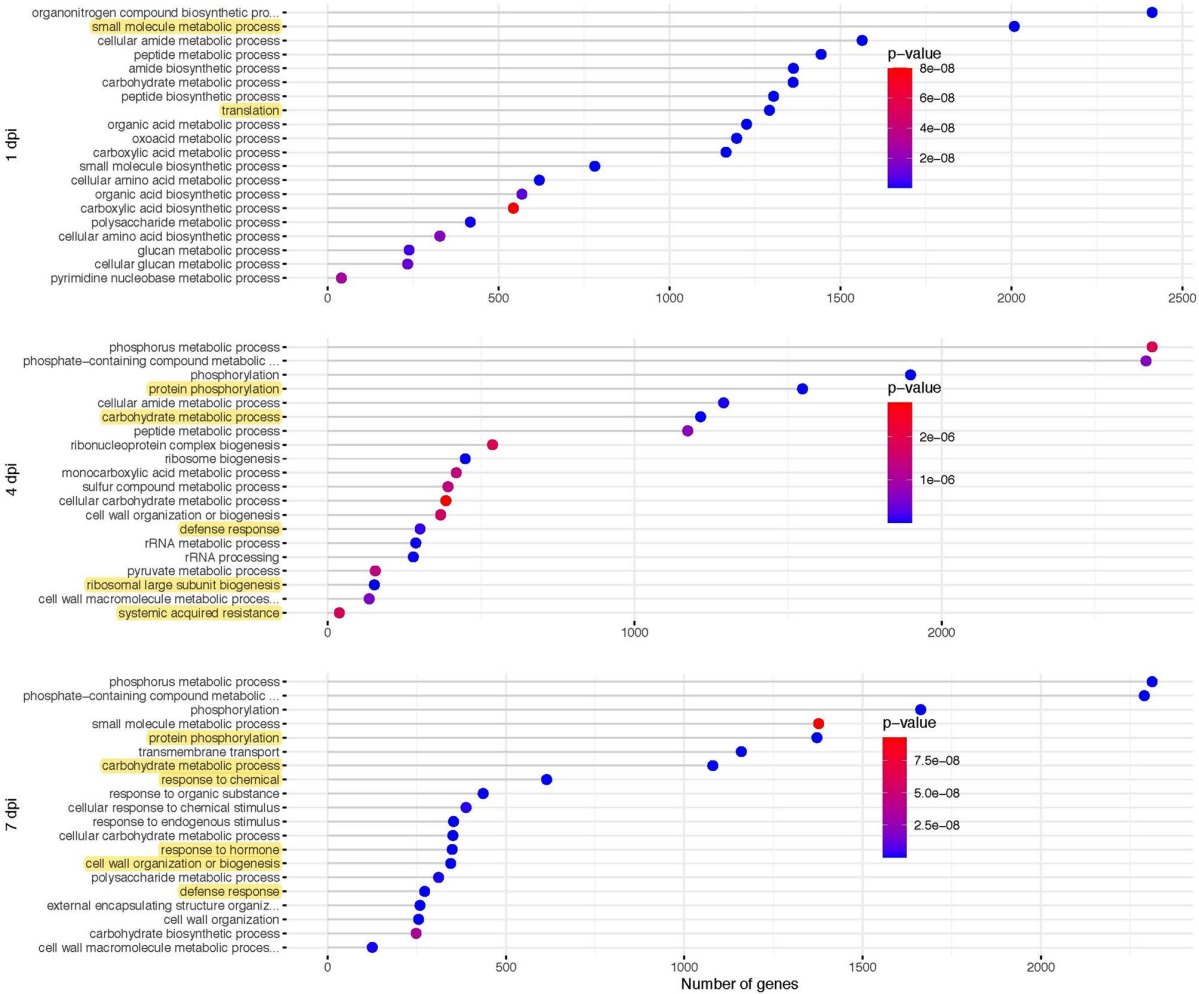
Gene ontology (GO) enrichment analysis of three different stages of colonisation using the biological process (BP) category. The number of genes associated with each GO term is shown on the x-axis and the specifically enriched GO terms for each time point are shown on the y-axis. Colour gradients represent the *P*-values for each of the GO terms. Highlighted text is mentioned in the results.

Different stages of colonisation varied for gene ontology (GO) terms that were enriched (Fig. 3). The most enriched biological process (BP) classifications during the initial infection stage at 1 dpi were translation and small molecule metabolic process, both in terms of the number of genes involved and the significance of the *P*-values. Protein phosphorylation became the most significantly enriched GO term at 4 dpi, although carbohydrate metabolic process and ribosomal large subunit biogenesis also featured strongly. The GO terms defence response and systemic acquired resistance (SAR) showed that at this stage the host reacted to the pathogen. There was an overlap in responses because at 7 dpi protein phosphorylation, carbohydrate metabolic process and defence response still featured. The *P*-value for the GO term cell wall organisation or biogenesis became more significant at 7 dpi than at 4 dpi. New GO terms at 7 dpi included response to chemical and response to hormone, indicating that at this later stage broad regulation of defence responses was initiated.

### Pathogen-responsive *WAKL10* expression is dependent on IL and temperature

Among 1646 DEGs, 733 and 913 of them were up- and down-regulated, respectively, with differences between Topas-*Rlm4* and Topas-*Rlm7* as a function of temperature (Table 1, Supplementary Table 1). A GO enrichment analysis was done on these 1646 DEGs (Supplementary Fig. 5). Vesicle-mediated transport was the most significantly enriched GO term of the BP category, but anion transport was also enriched. Virtually all GO terms of the cellular compartment (CC) category confirmed the importance of vesicle trafficking. The molecular function (MF) category confirmed these findings but also specified GO terms voltage-gated channel and mannan synthase activities.

**Figure 4:**
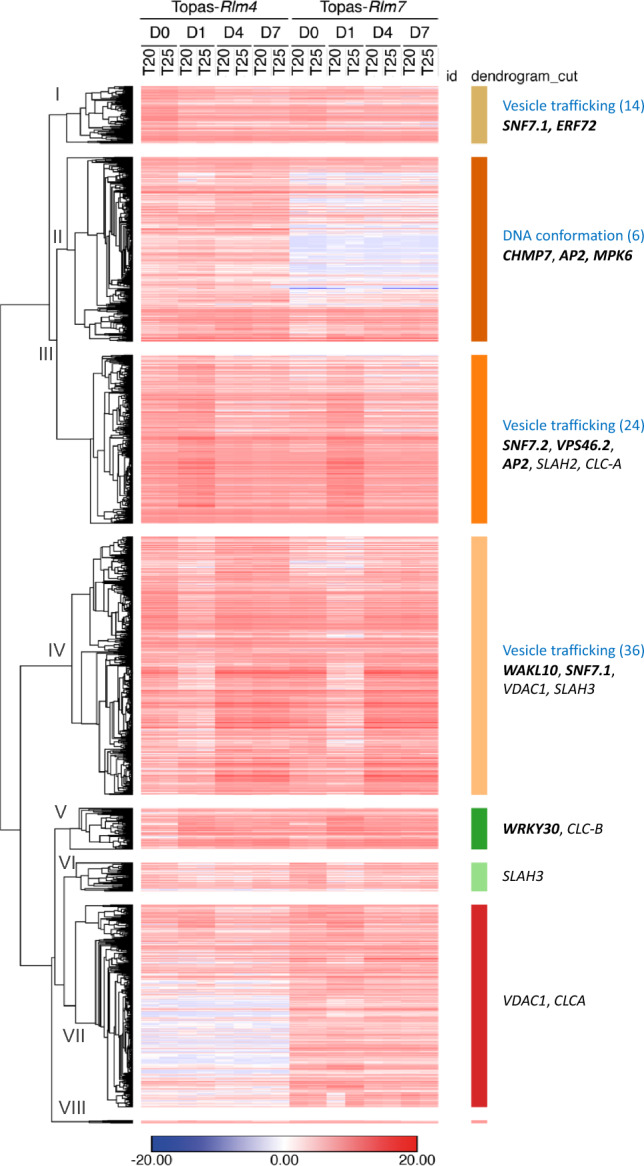
Heat map of 1646 differentially expressed genes (DEGs) that varied between Topas-*Rlm4* and Topas-*Rlm7* in a temperature-dependent fashion. Means of normalised expression values (n = 3) are shown according to the colour gradient. Hierarchical clustering of DEGs was done using one minus Pearson correlation with average as linkage method. Eight clusters were generated and illustrated using Roman numerals. The most significant gene ontology (GO) terms of the biological process (BP) category are listed for the first four clusters in blue because six or more DEGs represented the listed GO term (n ≥ 6, *P* < 0.002); numbers of DEGs belonging to the GO term are listed in parentheses. Clusters V to VIII had only two or one DEGs in the most significant GO term. DEGs of interest related to this study are highlighted in bold letters. *ERF72* contains an *APETELA2* (*AP2*)/B3-domain; *CHMP7* and *VPS46.2* have an *SNF7*-domain. *MPK6* refers to *mitogen-activated protein kinase 6*. *WAKL10* are *WALL ASSOCIATED KINASE-LIKE 10* genes. *SLAH2* and *SLAH3* refer to *SLOW ANION CHANNEL-ASSOCIATED 1* (*SLAC1*) homologs 2 and 3, respectively. *CLC-A* and *CLC-B* are chloride channel A and B genes, respectively. *VDCA1* refers to voltage dependent anion channel 1. DEGs related to ion transport are not highlighted in bold.

A heat map was generated and divided into eight clusters (Fig. 4). Each cluster was subjected to GO enrichment analysis and genes of interest were identified based on their annotations. Clusters I to IV comprised DEGs that were more highly expressed in Topas-*Rlm4* than Topas-*Rlm7* and vice versa for clusters V to VIII. Significantly, only the clusters I to IV had six or more DEGs belonging to the most significant GO term of the BP category, vesicle trafficking and DNA conformation change; clusters V to VIII had only one or two DEGs belonging to the most significant GO term of the BP category and were therefore not listed. Examples of defence-related and development-related DEGs expressed more highly in Topas-*Rlm4* than in Topas-*Rlm7* included *ERF72* (cluster I), the *SNF7*-domain containing *CHMP7*^40^ and *VPS46.2* (clusters II and III) and three *WAKL10* genes (cluster IV). The induction of two *WRKY30* genes (cluster V) at 1 dpi was more rapid in Topas-*Rlm4* than in Topas-*Rlm7* (Supplementary Fig. 6A). Three *WAKL10* genes (BnaA07g20220D, BnaC06g19670D, BnaC06g19690D) were more strongly induced and reached a greater level of expression at 4 dpi in Topas-*Rlm4* than in Topas-*Rlm7*; notable influences of line and temperature were observed. All three peptide sequences contain a highly conserved cytoplasmic domain (Supplementary Fig. 7), including the GC domain^24^. However, BnaC06g19690D lacks the galacturonan-binding WAK_GUB domain, and a calcium-binding EGF-like domain was identified only for BnaC06g19670D.

BAM files were used for sequence alignment and single nucleotide polymorphism (SNP) detection. Four of eight SNPs in Topas-*Rlm4* sequences of BnaA07g20220D resulted in amino acid substitutions (Supplementary Table 2). All three SNPs of Topas-*Rlm7* sequences of BnaA07g20220D yielded amino acid substitutions. Comparison of all sequence reads, including published information^30^, revealed a total of 11 non-synonymous and 6 synonymous SNPs, suggestive of positive selection. Detailed analysis of molecular evolution of three *B. napus*, two *B. oleracea*, one *B. rapa* and one *A. thaliana* sequences (Supplementary Fig. 8) provided evidence for significant positive selection of *WAKL10* genes located on *B. napus* chromosomes A07 and C06 (Supplementary Table 3).

### Temperature-sensitivity of resistance is regulated at the receptor level

Orthology information allowed inference of network interactions amongst *B. napus* genes. Union of regulatory (REG), protein–protein interaction (PPI) and KEGG networks resulted in a single (Uninet) network, which consisted of a total of 459,312 interactions with 51,581 REG, 396,319 PPI and 12,064 KEGG interactions. Only 652 interactions were shared between REG and PPI networks (Supplementary Table 4).

**Figure 5:**
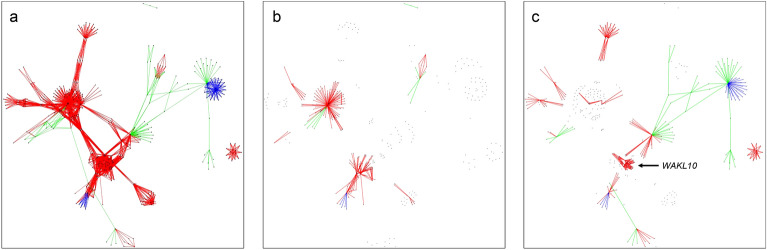
Gene-set (GS) sub-network archetypes. Red edges: protein–protein interactions (PPI); blue edges: regulatory interactions amongst transcription factors; green edges: KEGG interactions among genes and metabolites. (**a**) The GS sub-network was generated using 1,646 differentially expressed genes (DEGs) that were regulated in *Brassica napus* in response to *Leptosphaeria maculans* infection dependent on the Topas introgression line (IL) and temperature and a total of 459,312 network connections. This sub-network included all these connections. (**b**) Sub-network in Topas-*Rlm4* with connections influenced by temperature in this IL are shown. (**C**) Sub-network in Topas-*Rlm7* with connections influenced by temperature in this IL are shown. Note that a *WAKL10* PPI network occurs only in Topas-*Rlm7*.

The 1646 *B. napus* DEGs were combined with network information (Uninet) to create a sub-network (Fig. 5a). Two network archetypes were generated, one based on Topas-*Rlm4* (Fig. 5b; Supplementary Table 5) and another one based on Topas-*Rlm7* (Fig. 5c; Supplementary Table 6). Notably, a *WAKL10* PPI network was observed only in Topas-*Rlm7*, suggesting its involvement in temperature-sensitivity of *R* gene-mediated resistance against *L. maculans* in this IL.

Network topology metrics were generated using DEGs in Uninet (Supplementary Table 7), individually for Topas-*Rlm4* (Supplementary Table 8) and Topas-*Rlm7* networks (Supplementary Table 9). It was noticed that the overall network metrics showed imperceptible changes in Uninet, whereas significant differences in most metrics between the gene-set (GS) sub-networks of Topas-*Rlm4* and Topas-*Rlm7* were observed (Table 2, Fig. 5).

**Table 2:** Network archetypes topology overview.

Network	Topas-*Rlm4* network	Topas-*Rlm7* network	^1^GS sub-network Topas-*Rlm4*	GS sub-network Topas-*Rlm7*	Paired *t*-test *P*-value
^2^Average degree	26.92	26.85	12.31	11.66	0.0124
^3^Average betweenness	59,794.15	59,666.08	16.13	15.06	0.0007
^4^Average eigenvalues	0.01	0.01	0.13	0.1	0.0903
^5^Transitivity	0.02	0.02	0.15	0.16	0.0236
^6^Diameter	24	24	5	4	0.0163
^7^Average path length	7.06	7.06	1.84	1.84	N/A
^8^Assortivity degree	− 0.17	− 0.17	− 0.3	− 0.29	0.0255
Number of edges	450,767	448,881	2020	2105	0.0014
Number of vertices	33,477	33,427	328	361	0.0008

^1^Gene set (GS) sub-networks.

^2^The degree of a vertex or node is the number of adjacent edges.

^3^Vertex and edge betweenness are defined by the number of geodesics (shortest paths) going through a vertex or an edge; edges connecting separate modules have high edge betweenness as all the shortest paths from one module to another traverse through them.

^4^Eigenvector measures measures the influence of a node or vertex on a network.

^5^Transitivity or clustering coefficient measures the tendency of vertices or nodes to cluster together.

^6^The diameter of a graph is the length of the longest geodesic; determines the distance of the vertices that are furthest apart.

^7^Average unweighted shortest path length between all vertex pairs.

^8^The assortativity coefficient is positive is similar vertices tend to connect to each, and negative otherwise.

GO analysis using the network archetype information revealed a striking difference between the ILs. Whereas vesicle-mediated transport was the most significant GO-term of the Topas-*Rlm4* network, different GO-terms, including ion transport, were most significant and prominent for the Topas-*Rlm7* network (Supplementary Tables 10–15), implying a contribution of vesicle trafficking to temperature-resilient *R* gene-mediated resistance.

### Evidence for network propagation to defence-responsive transcription factors

**Figure 6:**
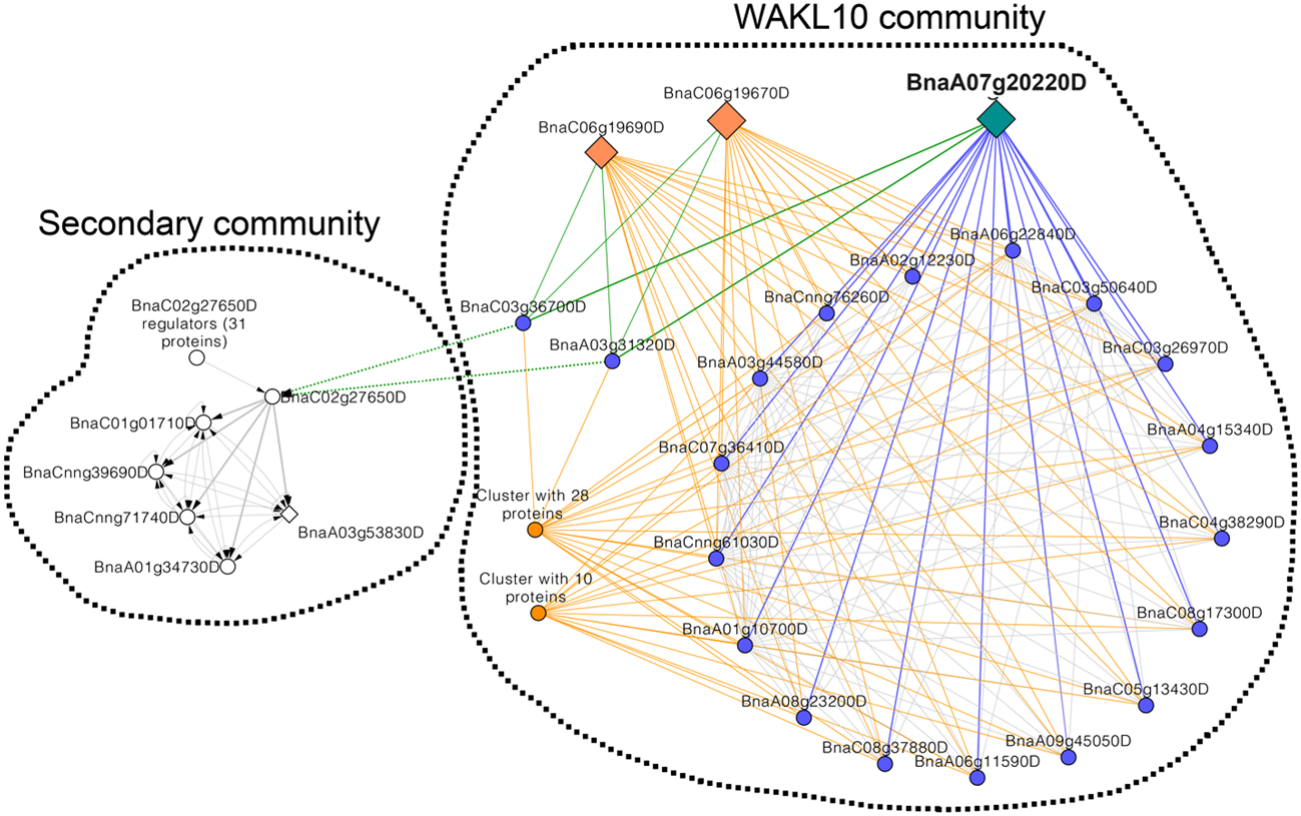
Network communities from *WAKL10* network diffusion analysis. Diamond nodes represent differentially expressed genes with size of the symbol related to the log_2_-fold change. The blue nodes represent proteins directly connecting *WAKL10* (BnaA07g20220D, BnaC06g19670D, BnaC06g19690D) to other proteins (orange and blue edges). The blue nodes include ubiquitin-conjugating enzyme 34 (BnaA06g11590D, BnaA08g23200D, BnaA09g45050D, BnaC08g37880D, BnaC08g17300D, BnaC05g13430D), IQ-domain 6 (BnaA04g15340D, BnaC04g38290D, BnaC03g26970D), LRR protein kinase family protein (BnaC03g50640D, BnaA06g22840D, BnaCnng61030D, BnaA01g10700D, BnaC07g36410D, BnaA03g44580D) and *NDR1*/*HIN1*-like gene *NHL6* (BnaCnng76260D, BnaA02g12230D). The green edges represent the main connections between the *WAKL10* community and a secondary community retrieved from the Topas-*Rlm4* network. The nodes connecting the green edges represent *SNF*-domain containing ESCRT-III complex-associated *VPS60.1* (BnaA03g31320D, BnaC03g36700D) that connects to *WRKY22* (BnaC02g27650). *WRKY22* further connects to *APETELA2*/*ETHYLENE RESPONSE FACTOR* (*AP2*/*ERF*)-type transcription factors (BnaC01g01710D, BnaCnng39690D, BnaCnng71740D, BnaA01g34730D, BnaA03g53830D).

Further analysis of the GS sub-networks showed that *WAKL10* (BnaA07g20220D, BnaC06g19670D and BnaC06g19690D) gene connections with its neighbours only occurred in Topas-*Rlm7* (Fig. 5). A network diffusion was generated to understand the role of *WAKL10* genes and their interactions in Uninet. The communities of the Topas-*Rlm7* network generated from the *WAKL10* network diffusion analysis had 97 nodes (Fig. 6, Supplementary Tables 16–18). Whereas all interactions among nodes in the *WAKL10* community of Topas-*Rlm7* represented PPIs, the Topas-*Rlm4* network consisted of gene regulatory interactions, including one gene (BnaA03g53830D) differentially expressed in response to *L. maculans*. Only three proteins identified in *WAKL10* community were encoded by genes that were differentially expressed; all represented *WAKL10* genes. We identified a single set of proteins connecting the *WAKL10* group to the rest of its community, including ubiquitin-conjugating enzyme 34, leucine-rich repeat (LRR) receptor kinases and IQ domain proteins. Sixteen of the 21 annotated genes encode transmembrane proteins (Supplementary Tables 16).

A secondary community was extracted from the Topas-*Rlm4* network. *SNF7*-domain containing and ESCRT-III complex-associated proteins *VPS60.1* (BnaA03g31320D and BnaC03g36700D) were physically connected to the *WAKL10* group and had regulatory interactions with *WRKY22* (BnaC02g27650D) in the secondary community (Fig. 6). Within the secondary community, *WRKY22* projected to five *APETELA2*/*ETHYLENE RESPONSE FACTOR* (*AP2*/*ERF*) genes. Two of these (BnaA01g34730D, BnaCnng71740D) lacked a start codon (Supplementary Table 18). One of the remaining genes (BnaC01g01710D) is more closely related to and in micro-synteny with *APETALA2* of *A. thaliana*, while the other two genes are related to other *B. rapa* (BnaA03g53830D) and *B. oleracea* (BnaCnng39690D) genes (Supplementary Fig. 9). *WRKY22* also had regulatory interactions with 31 proteins that included other immune regulators (Supplementary Table 17). The dynamics and interactions observed in the *WAKL10* community cannot be a direct by-product of signalling from the secondary community, suggesting that other, yet unidentified, genes regulate differential expression of *WAKL10* genes in response to *L. maculans* infection (Fig. 6). Indeed, phylogenetic footprinting of *WAKL10* promoter sequences^41^ identified several defence-responsive cis-acting elements, including putative W boxes, targeted by WRKY transcription factors, and Ca^2+^-responsive elements (Supplementary Fig. 10, Supplementary Table 19).

### Validation of pathogen-induced *PR1* expression

**Figure 7:**
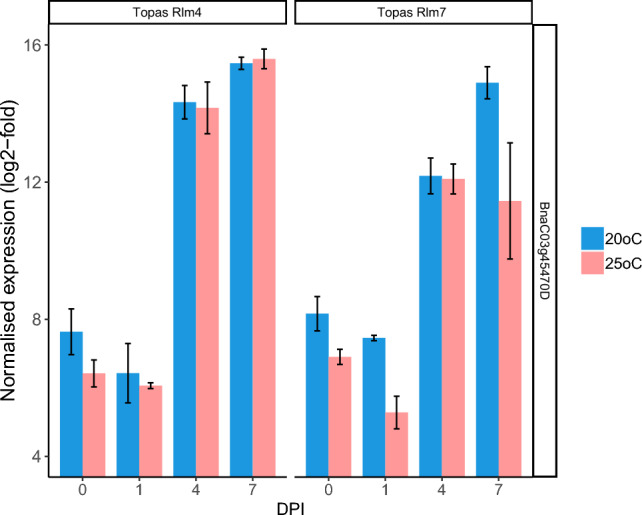
*Leptosphaeria maculans*-induced *PR1* (BnaC03g45470D) expression as evidenced using RNA-seq. Normalised expression was determined using DESeq2 and expressed as log_2_-fold. *Brassica napus* introgression lines Topas-*Rlm4* or Topas-*Rlm7* were inoculated with *Leptosphaeria maculans* isolate JN3 (*AvrLm1-4-5-6-7-8*) and incubated at 20 °C or 25 °C. Samples were taken at different days post-inoculation (DPI). Bar plots were generated in R with means and standard errors of the mean indicated. Three biological replicates per treatment were used.

*PR1* expression was used to determine the validity of the RNA-seq data. *PR1* was induced at 4 and 7 dpi (Table 1). Pathogen-induced *PR1* expression did not significantly differ between Topas-*Rlm4* and Topas-*Rlm7* and was not dependent on temperature (Fig. 7). A separate qPCR experiment was done to validate the RNA-seq data with Topas-*Rlm4* and Topas-*Rlm7* included in this analysis. Consistent with the RNA-seq experiment, *PR1* was identified as a gene induced at 4 and 7 dpi.

## Discussion

This work contributes to understanding temperature-sensitivity of *R* gene-mediated resistance against *L. maculans*. *LepR3* and *Rlm4* were identified as temperature-resilient *R* genes. While *Rlm2* appeared temperature-sensitive in cv. Bristol, resistance of Topas-*Rlm2* was temperature-resilient. *Rlm7* was temperature-sensitive in Topas-*Rlm7* and cv. Roxet but temperature-resilient in cv. Excel. This is significant because different alleles were reported for *Rlm7*^14^ and here shown to influence temperature-dependent *R* gene-mediated resistance.

Transcriptomics was used to better understand the influence of temperature on *R* gene-mediated resistance in Topas-*Rlm4* and Topas-*Rlm7*. Host transcriptomics was emphasised (i) because pathogen genes were not differentially regulated in response to line or line-by-temperature interaction and (ii) because axenic fungal growth was not influenced by temperature.

Host gene expression was different between mock-inoculated and infected plants at 1 dpi versus 4 and 7 dpi. An initial infection stage at 1 dpi was differentiated from defence response stages at 4 and 7 dpi. Specifically, SAR and response to hormone were enriched GO-terms for BP at 4 and 7 dpi, respectively. Protein phosphorylation featured strongly at 4 and 7 dpi, as previously documented^29,32^. Amongst up-regulated protein kinases, three *WAKL10* homologs were identified. The BnaA07g20220D gene, alleles of which encode *Rlm9*^16^, *Rlm4* and *Rlm7*, was previously found in a transcriptomics screen^29^. Detailed analysis of RNA-seq reads demonstrated that Topas-*Rlm7* harboured the *Rlm7-1* allele^14^, which we established to be temperature-sensitive. Donor parent cv. Roxet^39^ and cv. Excel therefore contain *Rlm7-1* and *Rlm7-2* alleles, respectively. Nine amino acid differences between *Rlm7-1* and *Rlm7-2* could be responsible for temperature-dependent differences; one of them resides in the intracellular domain of the receptor^14^.

A comparison of upstream regulatory sequences demonstrated conserved stretches in *WAKL10* promoter regions of BnaA07g20220D and BnaC06g19670D. The regulatory region upstream of the *B. napus* locus on chromosome A07 is undergoing diversifying sequence evolution, including gain of Ca^2+^-responsive elements in the BnaA07g20220D sequence of cv. Darmor *bzh*, which corresponds to *Rlm9*^16^.

A focussed search for genes regulated by temperature in an IL-dependent fashion revealed vesicle-mediated transport as the most significantly enriched GO-term across all categories. Amongst these genes were *SNF7*-domain containing genes *SNF7.1*, *SNF7.2* and the ESCRT-III complex-associated protein, *VPS46.2*^42^, which were more highly expressed in Topas-*Rlm4* than in Topas-*Rlm7*, encoding protein components of the plant-specific ESCRT-III complex^43^. The ESCRT-III complex generates intraluminal vesicles (ILVs) and multivesicular bodies (MVBs) that are destined for the plasma membrane to release exosomes or the vacuole^42^. Conversely, anion transporters were more strongly expressed in Topas-*Rlm7*. Collective analysis of these DEGs and network data revealed that vesicle trafficking was not a prominent and significant GO-term in Topas-*Rlm7*, suggesting that other processes, including membrane transport, are involved in the temperature-sensitive response of this IL no longer dedicated to *R* gene-mediated resistance at 25 °C. MVBs and exosome release are fundamental to penetration resistance against appressorium-forming filamentous pathogens as part of an evolutionarily ancient defence mechanism^44,45^. It is possible that similar mechanisms also contribute to defence against apoplastic fungal pathogens.

Network analysis demonstrated the appearance of a *WALK10* PPI network specifically in Topas-*Rlm7* at 25 °C. Amongst these interacting proteins was the ESCRT-III complex-associated protein *VPS60.1*. It is conceivable, albeit speculative, that the endosomal compartment facilitates an interaction between *WAKL10* and *VPS60.1* proteins for receptor trafficking in Topas-*Rlm7* at 25 °C. Ubiquitin-conjugating enzyme 34, which contributes to protein internalisation and sorting of cargo proteins, is another component of the *WAKL10* community^46^. Transmembrane proteins, *NHL6* and LRR receptor kinases of unknown function, were part of the *WAKL10* community core, as were IQ-domain proteins that are microtubule-targeted and contribute to microtubule organisation^47,48^. Collectively, these findings suggest that temperature-sensitivity in Topas-*Rlm7* is facilitated via vesicle trafficking, protein sorting and formation of ILVs at the endosome. Moreover, the *NHL6* and the LRR receptor kinases may be defence-related because the former is induced in response to SA and similar to non-race-specific disease resistance *NDR1* and harpin-induced *HIN1* genes, and the latter are involved in defence and/or development^48,49^. Their turnover and targeting to the vacuole or plasma membrane could conceivably down-regulate defence responses at the higher temperature in Topas-*Rlm7*. By analogy, sporophytic self-incompatibility breaks down at 29 °C relative to 23 °C, attributed to a reduction in complex glycan modification of the *S*-locus receptor kinase and retention in the endoplasmic reticulum rather than export to the plasma membrane^50^.

The other two clusters that were part of the *WAKL10* community included cysteine-rich receptor-like kinases, including *CRK22* that regulates defence responses, and an additional member of the LRR receptor kinase gene family^51^. Notably, *EPIDERMAL PATTERNING FACTOR LIKE 6* (*EPFL6*), a member of the cluster with 10 proteins, encodes a secreted peptide that has a temperature-dependent phenotype^52^. Additionally, a protein phosphatase 2C (AP2C2) was identified in the cluster with 28 proteins, which targets *MPK6*^53^, a kinase that is induced in resistant *B. napus* after *L. maculans* infection^30^; this finding suggests that *MPK6* temperature-dependent down-regulation in Topas-*Rlm7* may compromise ETD (Fig. 4, Supplementary Table 8).

The *WAKL10* community had regulatory interactions with a secondary community. *WRKY22* acts as the primary hub within this secondary community. This interaction may not be surprising because induction of *AtWAKL10* during leaf senescence down-regulates *AtWRKY22* expression and results in delayed senescence^35^. *WRKY22* is an important regulator of PTI downstream of MPK cascades like *MPK3*/*MPK6*^54^. *AtWRKY22* is linked to temperature responses with increased translation at 27 °C versus 17 °C and a hairpin structure in the 5’-UTR conferring responsiveness to increased temperature^55^. *AtWRKY22* is also involved in epigenetic control of defence responses against bacterial infection^56^, which may partially explain down-regulation of *WRKY22* expression after *L. maculans* infection in Topas-*Rlm7* in contrast to its stable expression in Topas-*Rlm4* (Supplementary Fig. 11).

*WRKY22* projected to a group of *AP2*/*ERF* genes. Although three of these genes are related full-length versions of the developmental *AP2* gene from *A. thaliana*, two of them may have diversified to acquire new roles in development, stress or pathogen responses^57,58^. *WRKY22* had regulatory interactions with 31 proteins, which corresponded to 11 genes in *A. thaliana*. All but one (BnaA02g23640D) were previously identified being targeted by *WRKY22* during submergence-induced immunity^59^. WRKY22 was also reported to target the *VPS60.1* promoter, providing support for the link between *WAKL10* and the secondary community.

To conclude, increasing temperature is one the most important abiotic factors shaping plant–microbe interactions. Temperature-sensitive *R* gene-mediated resistance is of concern because global warming may lead to new disease epidemics. Identification and characterisation of temperature-sensitive *R* genes are important for breeding resilient crops. The temperature-sensitive *Rlm7-1* gene was unable to induce vesicle-mediated defence signalling at 25 °C. Instead, the *Rlm7*-encoded WAKL10 receptor may be targeted to intraluminal vesicles that are destined for the vacuole or the cell wall via exosome release. In either case, this WAKL10 receptor will then no longer be available for signalling defence. Instead, it might scavenge *AvrLm4-7* effectors in the cell wall that will no longer be recognised at the plasma membrane. This provides a novel model for temperature-sensitive *R* gene-mediated resistance against apoplastic fungal pathogens.

## Methods

### Genetic plant material and pathogen isolates

Cotyledon inoculation assays at 20 °C and 25 °C were done with *L. maculans* isolates JN3 (*AvrLm1-4-5-6-7-8*) and 99–79 (*AvrLm2-4-7*) to identify temperature-sensitive and resilient *R* genes from a set of *B. napus* ILs, containing *Rlm2, Rlm4, Rlm7* or *LepR3* introgressed into a Topas DH16516 background, developed at the Saskatoon Research and Development Centre of Agriculture and Agri-Food Canada^39^. *B. napus* cultivars containing the same set of *R* genes were tested; cv. Bristol (*Rlm2*), cv. Surpass (*LepR3*), cv. Adriana (*Rlm4*), cv. Excel (*Rlm7*) and cv. Roxet (*Rlm7*)^60,61^. Cultivars Bristol, Surpass, Adriana and Excel are commonly used as a differential set to determine *Avr* genes in *L. maculans* isolates^62^. Cultivar Roxet was the source of *Rlm7* introgressed into Topas. *Rlm7* in cv. Excel originates from cv. Caiman^14^. All experimental plant research complied with relevant institutional, national and international guidelines and legislation.

To prepare inoculum, selected *L. maculans* isolates were sub-cultured on V8 agar plates for pycnidial development and asexual sporulation^12,63^. Conidia were harvested from sporulating plates to make conidial suspensions, adjusted to 10^7^ ml^−1^ and stored at − 20 °C.

### Plant growth and inoculation of cotyledons

Seeds were sown in 50-cell trays filled with a 1:1 ratio of John Innes number 3 and Miracle-Gro compost. Plants were grown in controlled environment chambers with a 12-h light/12-h dark cycle at 20 °C and a relative humidity of 70%. Light intensity was 320 µmol m^−2^ s^−1^. Twenty-four hours before inoculation, plants were divided into one group remaining at 20 °C and the other group moved to 25 °C with other conditions kept the same^12^.

Ten-days old *B. napus* cotyledons were point-inoculated as described^63^. Ten µl of conidial suspension were placed onto each wound site. Following inoculation, seedlings were kept in the dark for 24 h. True leaves were removed to allow full expansion of cotyledons and prevent senescence. Disease assessment was done 12 dpi on a 0–9 scale^39^.

### Effect of temperature on growth rate of L. maculans isolates

Isolates JN3 and 99–79 were grown at 20 °C or 25 °C in darkness, placing mycelial discs (6 mm diameter), taken from perimeters of agar-grown cultures, upside down in the centre of V8 agar Petri plates^12^. Mycelial growth rates were compared by measuring the diameter of each culture.

### Sample preparation for transcriptomics

RNA-seq analysis was done at 0, 1, 4 and 7 dpi with cotyledons of Topas-*Rlm7* or Topas-*Rlm4* inoculated with isolate JN3 to examine gene expression at 20 °C and 25 °C. Four leaf discs (8 mm diameter) were taken from seedlings (two leaf discs per cotyledon), and five seedlings were sampled per treatment using a sterile Rapid-Core biopsy punch (ProSciTech). Samples were placed in 2 ml screw-cap Eppendorf tubes with a zirconium oxide (5 mm) grinding ball (Retsch) and submerged in liquid nitrogen. Frozen tissue was ground into a fine powder using a Mixer Mill MM 400 (Retsch). An E.Z.N.A. Plant RNA Kit (Omega Bio-tek) was used to extract RNA following the manufacturer’s protocol. RNase-free DNase I Set (Omega Bio-tek) was used to remove DNA contamination. Eluted RNA from each sample was divided into three aliquots for quantification, quality checking or RNA-seq.

RNA was quantified using a Nanodrop ND-1000 spectrophotometer (NanoDrop Technologies, Inc.) and a Qubit 3 fluorometer (Invitrogen). Of five replicates with the greatest concentration, four replicates with the greatest RNA quality were analysed on an Agilent 2100 Bioanalyzer (Agilent Technologies) using an RNA Nano Assay (Agilent Technologies) at Warwick Genome Facility. Three replicates with the greatest RNA integrity (RIN) values were submitted to GENEWIZ (South Plainfield, New Jersey, USA) for library preparation and RNA-seq.

### RNA-seq data analysis

A total of 48 RNA samples were sent for poly-A^+^ RNA selection and sequencing using Illumina HiSeq 150 bp paired-end configuration. Resulting fastq files were checked using FastQC. Adapters and barcodes were removed with Trimmomatic. *HISAT2* was used to map reads for each RNA-seq sample to *B. napus* AST_PRJEB5043_v1 and *L. maculans* URGI-INRA reference genomes. Transcript abundance was estimated with Bioconductor package FeatureCounts and further analysed using DESeq2 version 1.28.1^64^.

Plant and fungal datasets were analysed using a multi-factorial design with time, line, temperature and their interactions as factors. Transcripts with counts < 1 for all samples were removed from the datasets. Transcript counts were normalised to identify DEGs. PCA charts were done to visualise variation between samples using the R package ggplot2. Unless mentioned otherwise, a false discovery rate (FDR) of α = 0.05 was used to determine and visualise DEGs.

The UpSetR package was used to visualise intersections between DEGs. Heat maps were generated using Morpheus (https://software.broadinstitute.org/morpheus). Hierarchical clustering of rows (genes) was done using one minus Pearson correlation with average as linkage method. Dendrogram cuts were generated by sliding the threshold line to the left of the dendrogram rightwards until a reasonable number of clusters was identified. The software IGV 2.11.2 was used for alignment visualisation of RNA-seq reads to the reference genome. BAM files were used for detection of SNPs, insertions or deletions in Topas-*Rlm4* and Topas-*Rlm7*.

### Gene ontology enrichment analysis

The R package TopGO was used to compare DEGs to the *B. napus* dataset within Ensembl Plants. BP, CC and MF categories were used. Fisher’s exact test was used to calculate *P*-values and the topNodes parameter was set to 20 to return the most significant GO terms. The top 20 GO terms with the number of significant genes and *P*-values per term were visualised using Lollipop charts in R.

### Phylogenetic analysis

Phylogenetic analysis by maximum likelihood (PAML) was used^65^. CODEML was used to compare different models, including a beta model with two free parameters and beta & ω model with two additional parameters, one of which relates to the ratio of nonsynonymous/synonymous substitution rates (ω = dN/dS) with ω > 1 for positive selection. Likelihood ratio tests and posterior probabilities were computed. Bayes empirical Bayes (BEB) estimates were used to identify positively selected amino acid residues^66^.

CLC Sequence Viewer 8 and Geneious 10.0.9 were used to generate alignments of amino acid and upstream regulatory nucleotide sequences, respectively. The PLACE database was used to identify promoter motifs.

### Network inference

The transcription factor (TF) network was obtained from Arabidopsis Gene Regulatory Information Server (AGRIS)^67^⁠; only “confirmed interactions” from AtRegNet dataset were kept and considered as the REG network. The PPI network of *Arabidopsis thaliana* was obtained from Biogrid^68^; PPI data were filtered to keep *A. thaliana* non-redundant interactions confirmed by affinity chromatography technology (MI:0004), X-ray crystallography (MI:0114), far western blotting (MI:0047), fluorescent resonance energy transfer (MI:0055), protein complementation (MI:0090), experimental interaction detection (MI:0045) and yeast two-hybrid (MI:0018) experiments.

The g:Orth tool of g:Profiler^69^⁠ was used to obtain *B. napus* orthologs of *A. thaliana* genes. Only genes with orthologs in *B. napus* were kept. KEGG data^70^ for *B. napus* were downloaded via REST API to create a gene-compounds network. All networks were merged into one (Uninet). Visualisation of network analysis was done using the R package igraph^71^⁠.

### Inference of network archetypes

To create network archetypes, DESeq2 was used to obtain DEGs by evaluating all conditions (line, temperature and day). Transcripts with a false discovery rate (FDR/*P*_*adj*_) < 0.01 were kept. The g:Profiler^72^ was used for GO enrichment using *A. thaliana* orthologs.

Two network archetypes were generated following established methodology^73^. Genes without differential expression and interactions present in Uninet were used as the network base state. Down-regulated genes were added to create the Topas-*Rlm4* network (base state of comparison considering the interaction between line, temperature and day). The Topas-*Rlm7* network excluded down-regulated genes and their interactions, while up-regulated genes and their interactions were added. Sub-networks of the respective archetypes were generated from Uninet based on the list of DEGs.

### Network diffusion analysis

The most relevant genes/proteins affected by *WAKL10* in the network were assessed. A sub-network with a community containing BnaA07g20220D and its two closest neighbours from the Topas-*Rlm7* network archetype was created. The information flow from *WAKL10* homologs throughout that sub-network was calculated with Diffusion algorithm^74^⁠. The top 1% of the ranked nodes (60 proteins) from diffusion analysis was selected. Another community was created by searching in the Topas-*Rlm4* network archetype including 60 proteins of the previous community with their two closest neighbours.

### Reporting summary

Further information about research design is available in the Nature Portfolio Reporting summary linked to this article.

### Supplementary Information

Supplementary Information 1.

Supplementary Information 2.

Supplementary Information 3.

Supplementary Information 4.

Supplementary Information 5.

Supplementary Information 6.

Supplementary Information 7.

Supplementary Information 8.

Supplementary Information 9.

Supplementary Information 10.

Supplementary Information 11.

Supplementary Information 12.

Supplementary Information 13.

Supplementary Information 14.

Supplementary Information 15.

Supplementary Information 16.

Supplementary Information 17.

Supplementary Information 18.

## Data Availability

The sequence data analysed in this study will be available from the NCBI Bioproject database (accession: PRJNA1018845) following publication, as Biosamples SAMN37674651-SAMN37674698, SRR34352944-SRR34352991.

## Code availability

Although standard code was used for all analyses, code will be made available upon request.
