# Supplementary material for: Transcriptomics of temperature-sensitive R gene-mediated resistance identifies a WAKL10 protein interaction network
Source: Sci Rep. 2024 Feb 29;14:5023. doi: 10.1038/s41598-024-53643-7 (PMC10904819; doi:10.1038/s41598-024-53643-7)
Supplement: Supplementary file 1 — Supplementary Information 1. [file 41598_2024_53643_MOESM1_ESM.pdf]

Supplementary Fig. 1

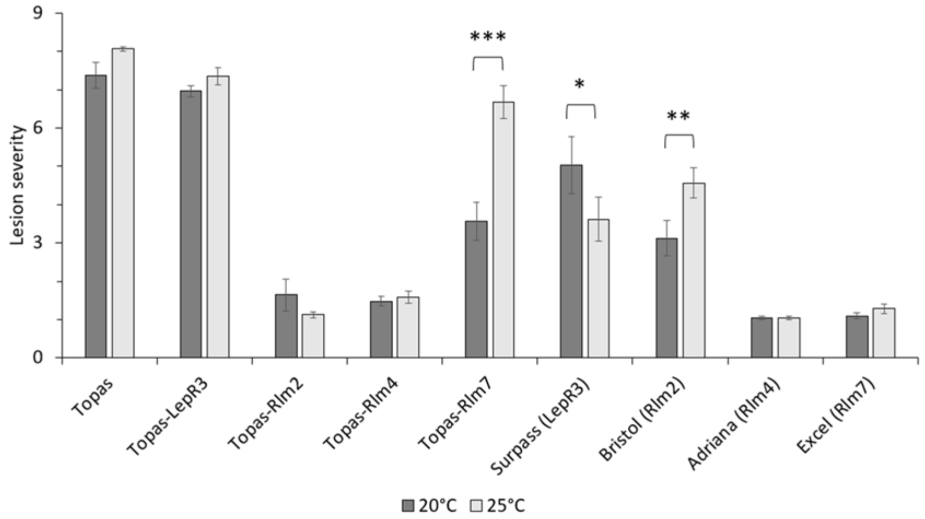

Supplementary Fig. 1 | Average lesion score, 0 (resistant) to 9 (susceptible) scale, assessed on cotyledons of *B. napus* Topas introgression lines, containing *LepR3*, *Rlm2*, *Rlm4* or *Rlm7*, and a differential set of cultivars containing each of these *R* genes, following wound inoculation with 10 µl of 10^7 ml^{-1} conidial suspension of *L. maculans* isolate 99-79 (*AvrLm2-4-7*) at 13 dpi at 20ºC or 25ºC. Four wound sites were assessed per plant. Eight biological replicates were included for each of the introgression lines and six biological replicates were included for each the differential set of cultivars for each assay, and each assay which was done twice. Bars represent mean lesion score and error bars indicate the standard errors of the mean (* $P < 0.05$, ** $P < 0.01$, *** $P < 0.001$).

Supplementary Fig. 2

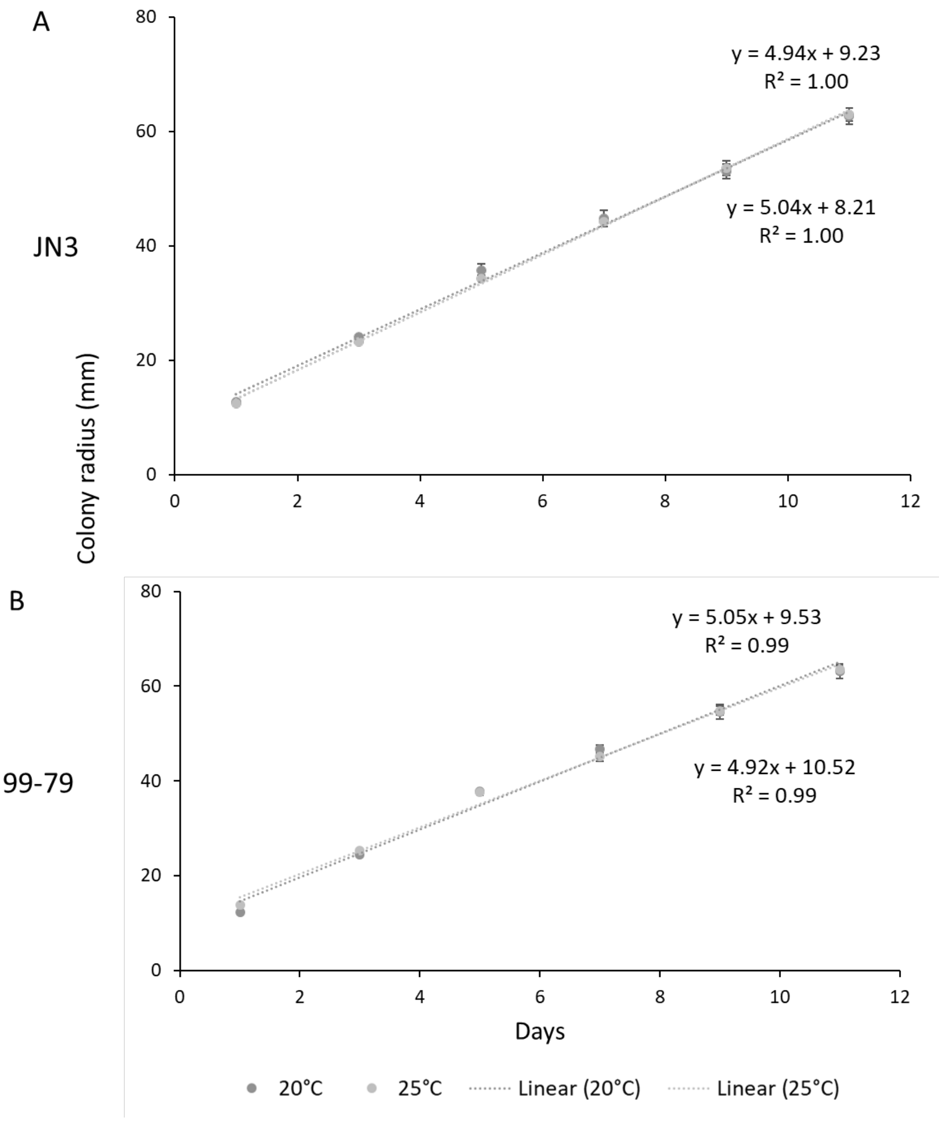

Supplementary Fig. 2 | Increased temperature from 20°C to 25°C had no effect on the radial growth of *L. maculans* isolates (A) JN3 (P = 0.26) or (B) 99-79 (P < 0.66). Mycelial discs were transferred from fungal colonies onto V8 media Petri dishes and placed in controlled environment cabinets set to constant temperatures of 20°C or 25°C. Colony diameter in mm was determined over a period from 1 to 11 days post-inoculation. Means and the standard errors of the mean are shown.

Supplementary Fig. 3

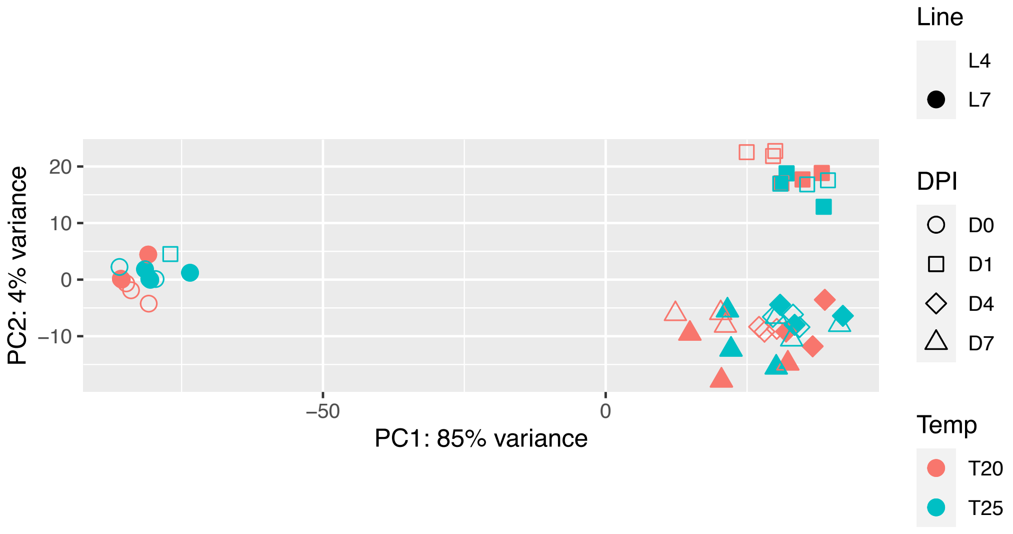

Supplementary Fig. 3 | Principal component (PC) analysis of RNA-Seq samples. *Brassica napus* introgression lines Topas-*Rlm4* or Topas-*Rlm7* were inoculated with *Leptosphaeria maculans* isolate JN3 (*AvrLm1-4-5-6-7-8*) and incubated for 0, 1, 4 and 7 days at 20ºC or 25ºC. Fungal transcriptome data were separated by genotype T-*Rlm4* and T-*Rlm7* (open versus filled symbols), temperature 20ºC and 25ºC (red versus blue outlines) and time 0, 1, 4 and 7 dpi (different shapes).

Supplementary Fig. 4

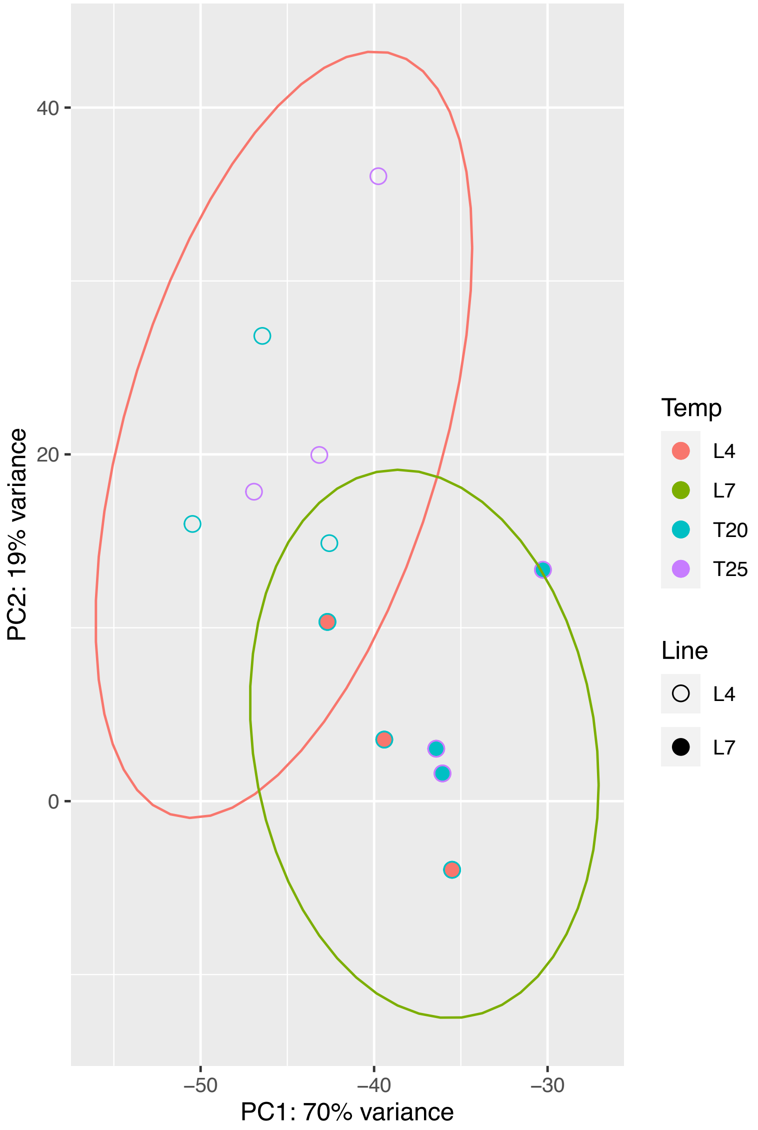

Supplementary Fig. 4 | Principal component (PC) analysis of RNA-Seq samples. *Brassica napus* introgression lines Topas-*Rlm4* or Topas-*Rlm7* were inoculated with *Leptosphaeria maculans* isolate JN3 (*AvrLm1-4-5-6-7-8*) and incubated for 4 days at 20ºC or 25ºC. Host transcriptome data were separated by genotype T-*Rlm4* and T-*Rlm7* (open versus filled symbols), temperature 20ºC and 25ºC (blue versus purple outlines). Red and green ellipses outline areas of 95% confidence for T-*Rlm4* and T-*Rlm7*, respectively.

Supplementary Fig. 5

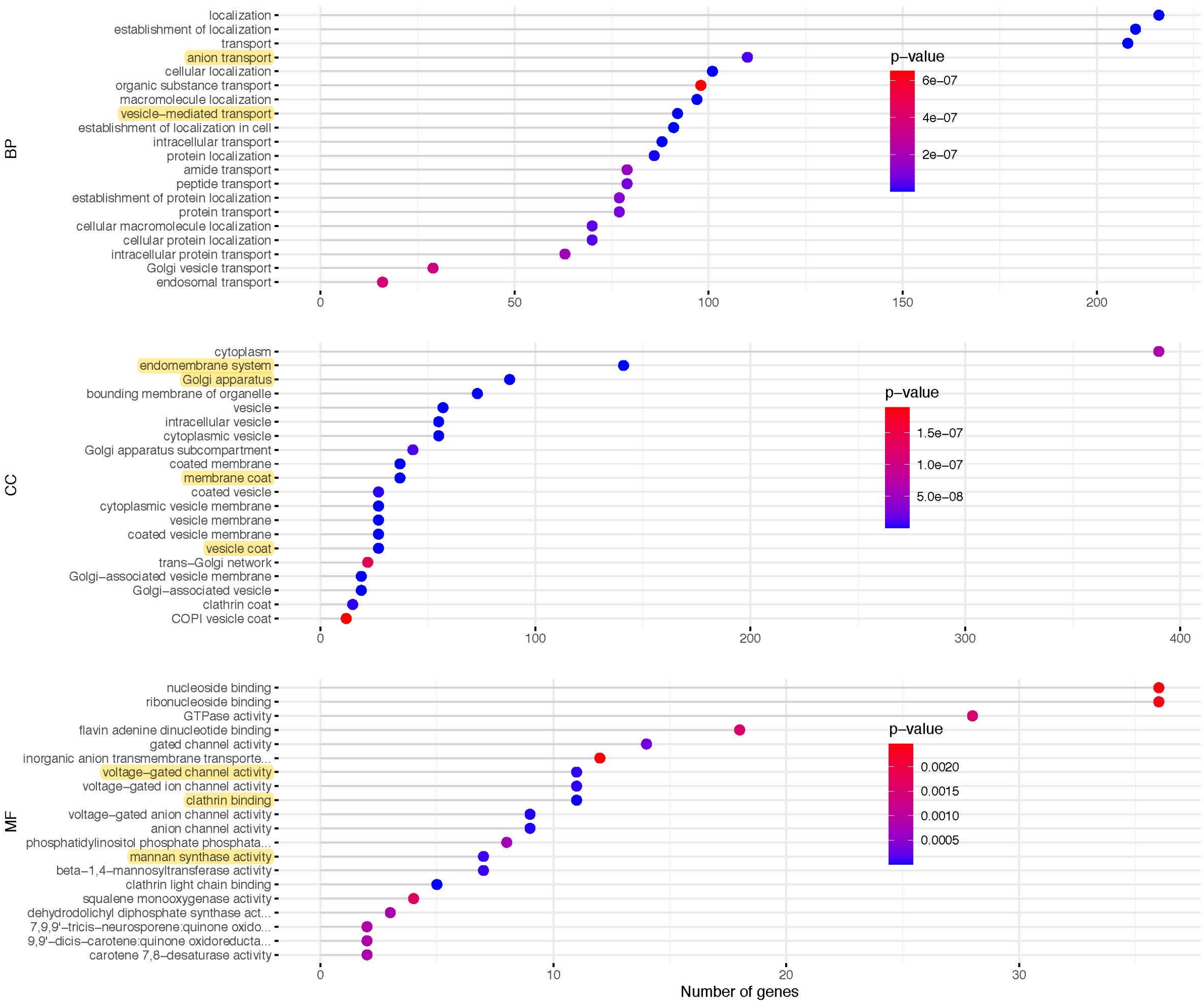

Supplementary Fig. 5 | Gene ontology (GO) enrichment analysis of differentially expressed genes (DEGs) that varied between genotypes Topas-*Rlm4* and Topas-*Rlm7* in a temperature-dependent fashion after infection with *Leptosphaeria maculans*. Biological process (BP), cellular compartment (CC) and molecular function (MF) categories were considered. The numbers of genes associate with each GO term are shown on the x-axis and the specifically enriched GO terms for each GO category are shown on the y-axis. Colour gradients represent the *P*-values for each of the GO terms. Highlighted text referring to highly significant GO terms ($P < 10^{-4}$) is mentioned in the text of the paper.

Supplementary Fig. 6

a

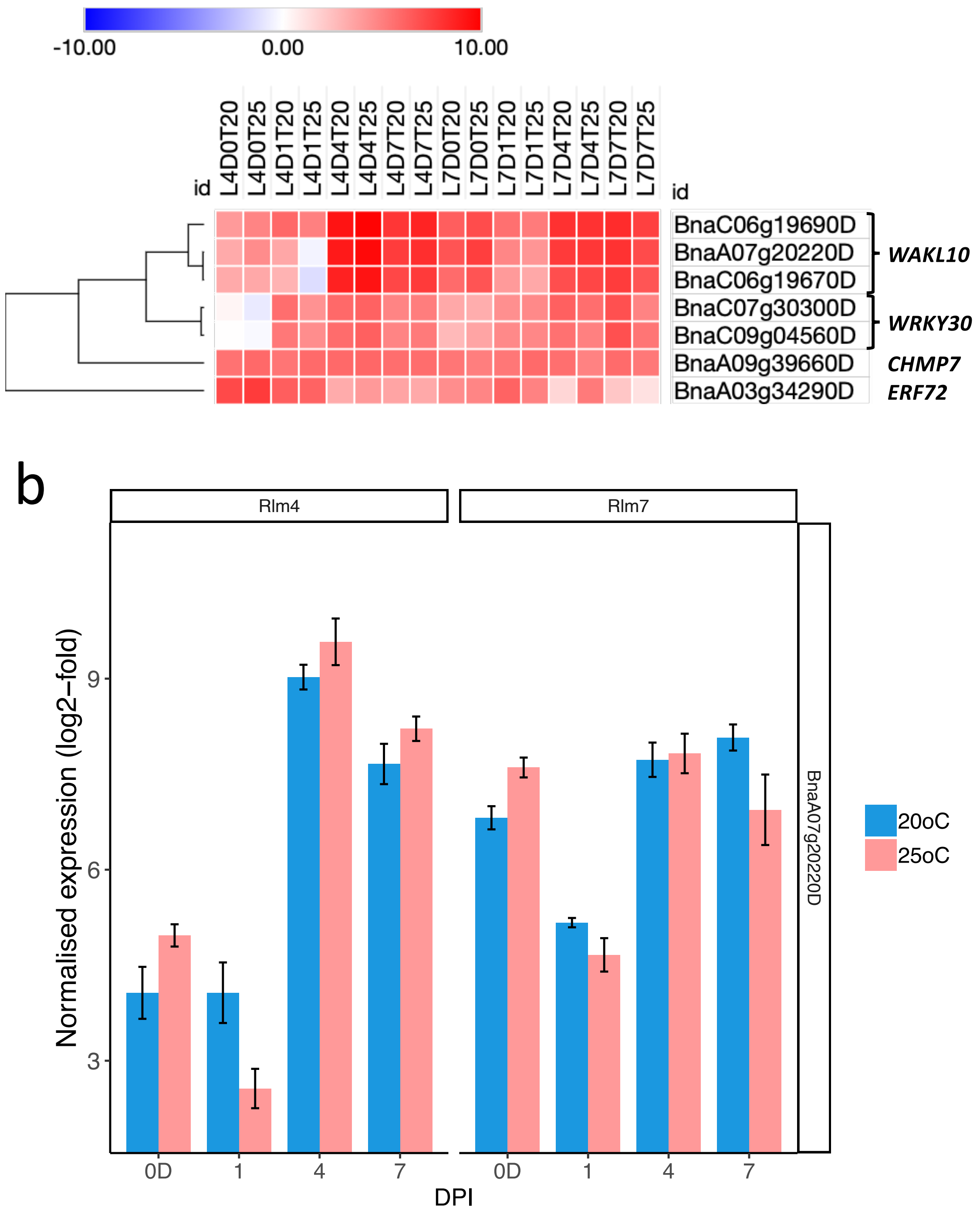

Supplementary Fig. 6 | Expression of selected *Brassica napus* genes in response to *Leptosphaeria maculans* infection that were influenced by temperature. (**a**) Heat map of subsets of differentially regulated genes (DEGs). Topas-*Rlm4* (L4) and Topas-*Rlm7* (L7) introgression lines were analysed at 0 dpi (D0), 1 dpi (D1), 4 dpi (D4) and 8 dpi (D8) upon incubation at 20°C (T20) or 25°C (T25). Means of normalised expression values are shown according to the colour gradient. Hierarchical clustering of DEGs was done using one minus Pearson correlation with average as linkage method. ERF72 and CHMP7 are functionally characterised genes containing APETELA2 (AP2)/B3-domains and SNF7-domains, respectively. *WAKL10* are *WALL ASSOCIATED KINASE-LIKE 10* genes. *WRKY30* genes encode defence-related transcription factors. These genes were selected from a more comprehensive analysis shown in Figure 4. (**b**) Bar plot of the *WAKL10* gene BnaA07g20220D expression based on normalised $\log_2$-transformed RNA sequencing data using DESeq2. Error bars indicate standard errors of the mean (n = 3).

Supplementary Fig. 7

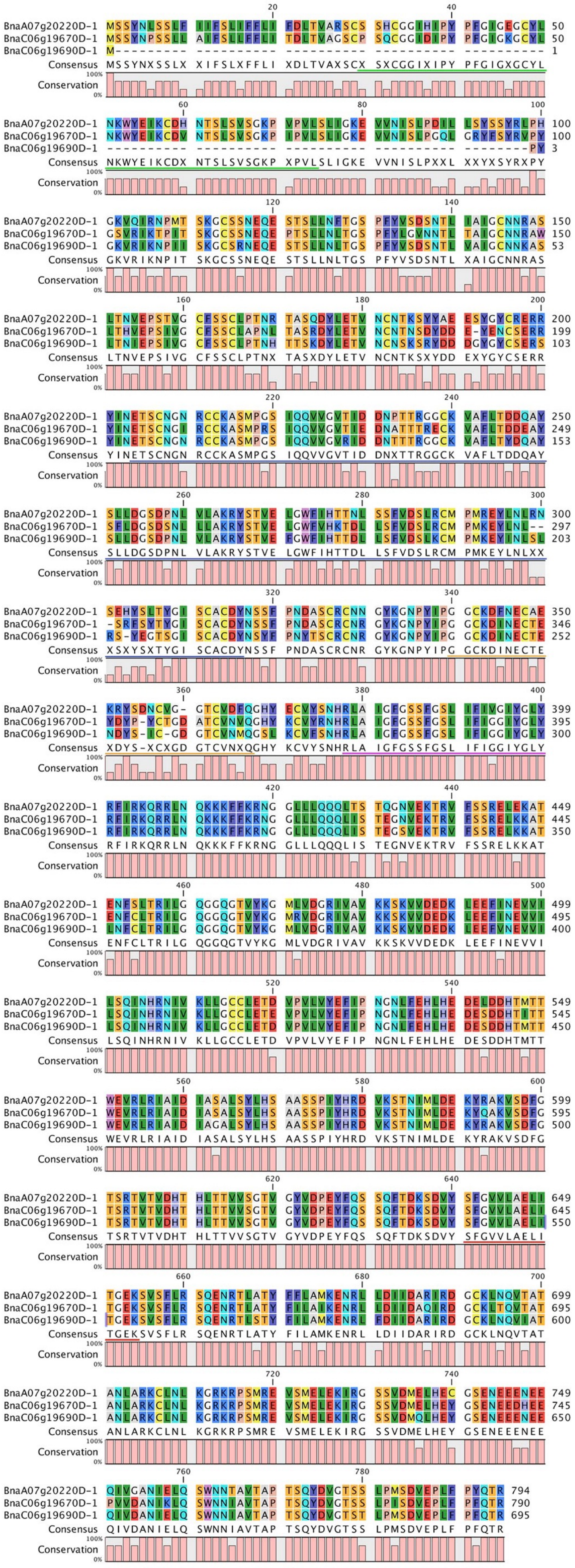

Supplementary Fig. 7 | Alignment of *WAKL10* amino acid sequences. Domains of interest are underlined in green (galacturonan-binding; WAK_GUB), blue (WAK), orange (calcium-binding EGF-like; IPR018097), purple (transmembrane helix) or red (guanylyl cyclase). Note insertion-deletion polymorphisms that affect the extracellular but not the cytoplasmic domain.

Supplementary Fig. 8

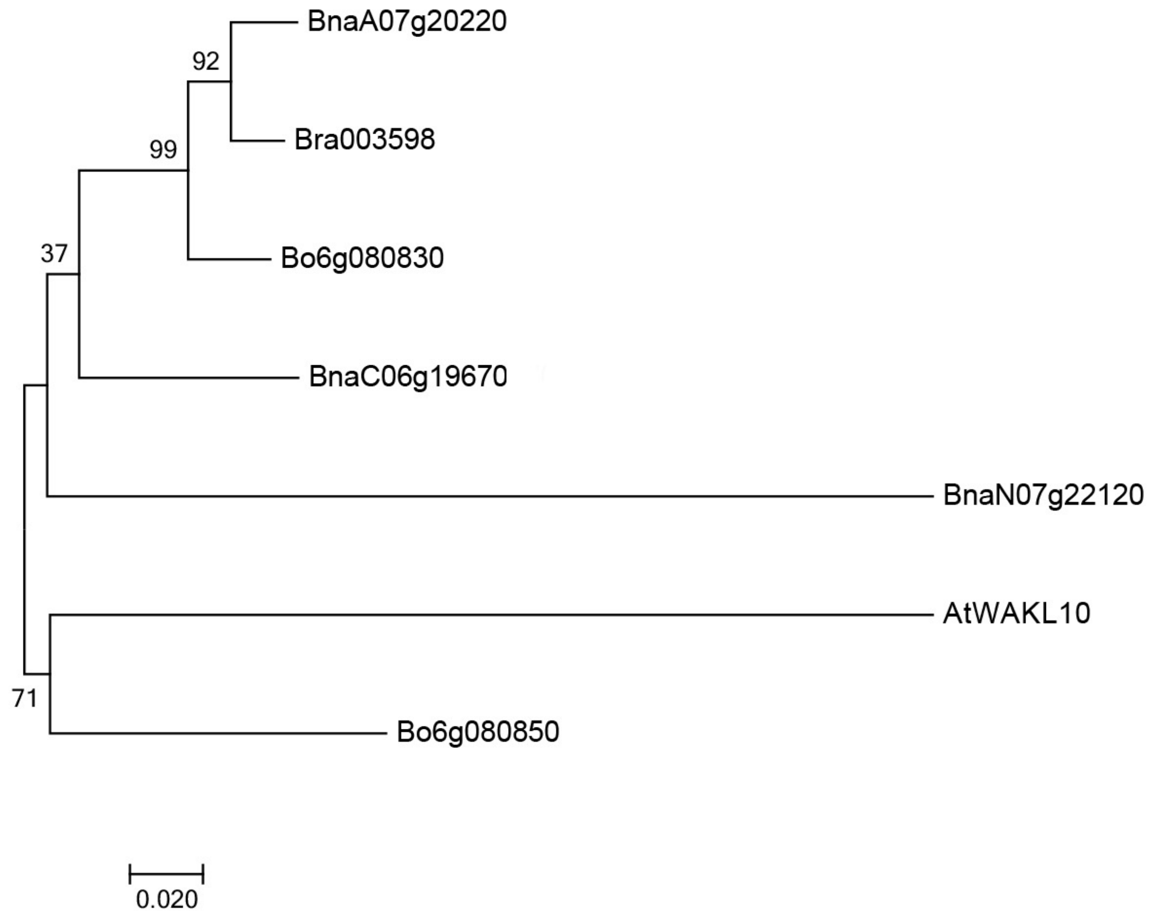

Supplementary Fig. 8 | Maximum likelihood phylogenetic tree of *WALL ASSOCIATED KINASE-LIKE (WAKL) 10* genes. Coding sequences were used to generate the tree. The Jukes-Cantor model [28] was used. The tree with the greatest log-likelihood is shown. Numbers indicate bootstrap values of 1,000 replicates. A discrete Gamma distribution was used to model evolutionary rate differences between sites. The rate variation model allowed for some sites to be evolutionarily invariable. Branch lengths are measured as the number of substitutions per site (scale bar). Branches designate species abbreviations: Bna = *Brassica napus*, Bra = *B. rapa* and Bo = *B. oleracea* and At = *Arabidopsis thaliana*.

Supplementary Fig. 9

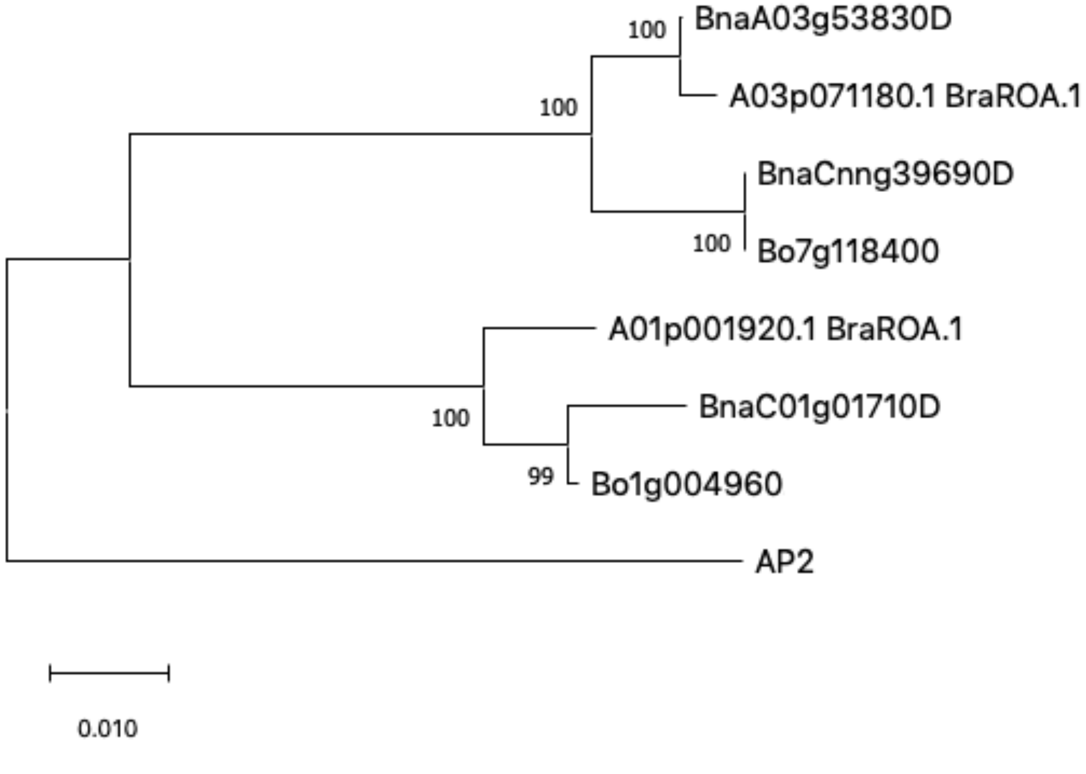

Supplementary Fig. 9 | Maximum likelihood phylogenetic tree of *APETALA2* genes. Coding sequences were used to generate the tree. The Tamura-Nei model was used. The tree with the greatest log-likelihood is shown. Numbers indicate bootstrap values of 500 replicates. Evolutionary rates did not vary between sites. Branch lengths are measured as the number of substitutions per site (scale bar). Branches designate species abbreviations: Bna = *Brassica napus*, Bra = *B. rapa* and Bo = *B. oleracea. AP2* is from *Arabidopsis thaliana*.

Supplementary Fig. 10

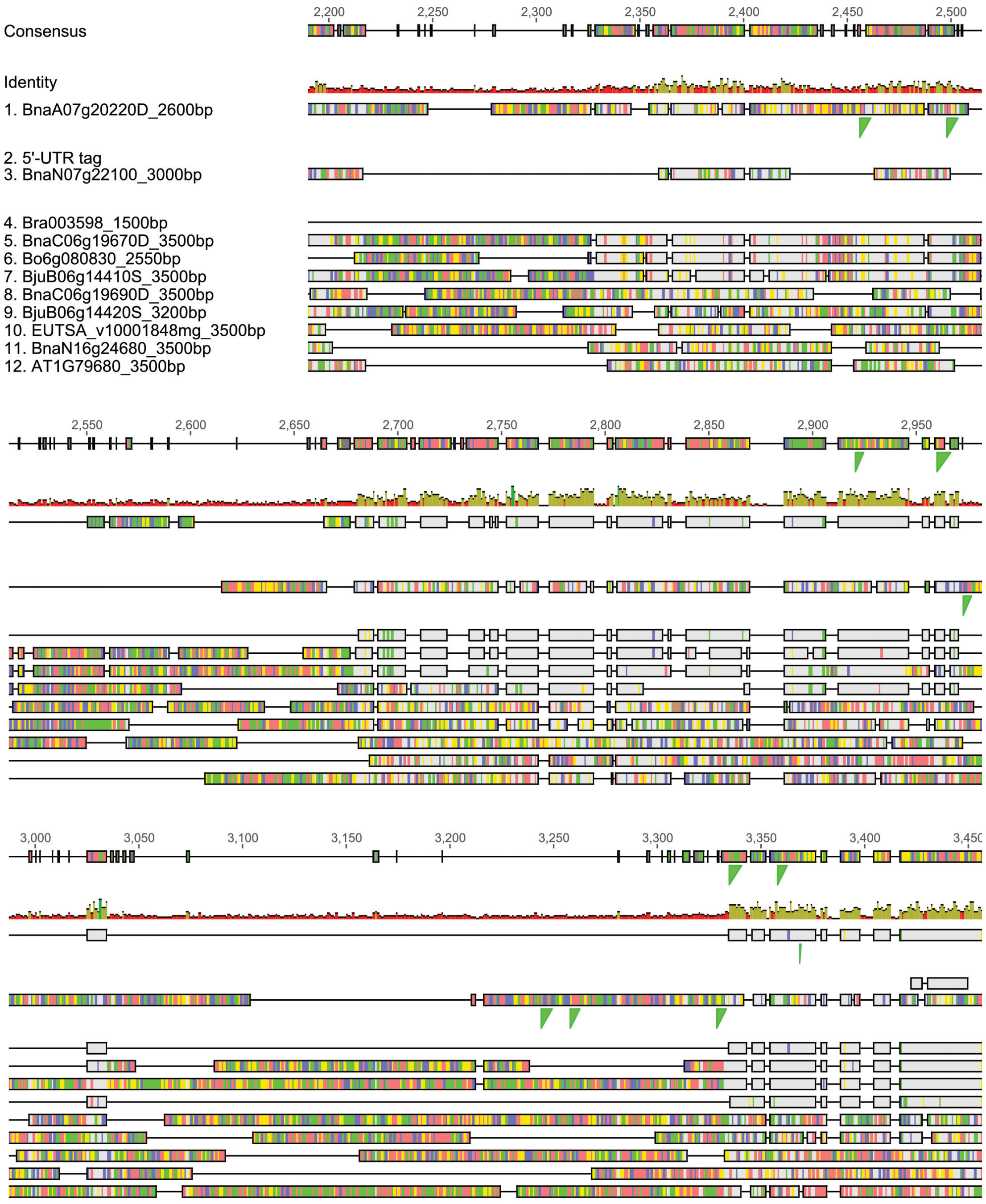

Supplementary Figure 10 | Alignment of promoter sequences of 11 wall-associated kinase-like genes. The consensus sequence is illustrated on the top with the nucleotide identities below. Grey boxes illustrate sequence identity. Nucleotides are colour-coded red (A), green (T), yellow (G) or blue (C). The 5'-UTR tag was identified using bam files of the transcriptome in the *Brassica napus* background Topas DH. Putative transcriptional start sites, TATA box and cis-acting transcription factor binding sites are indicated with green triangles. This image was generated with Geneious software. Information about these sites can be found in Table S9.

Supplementary Fig. 11

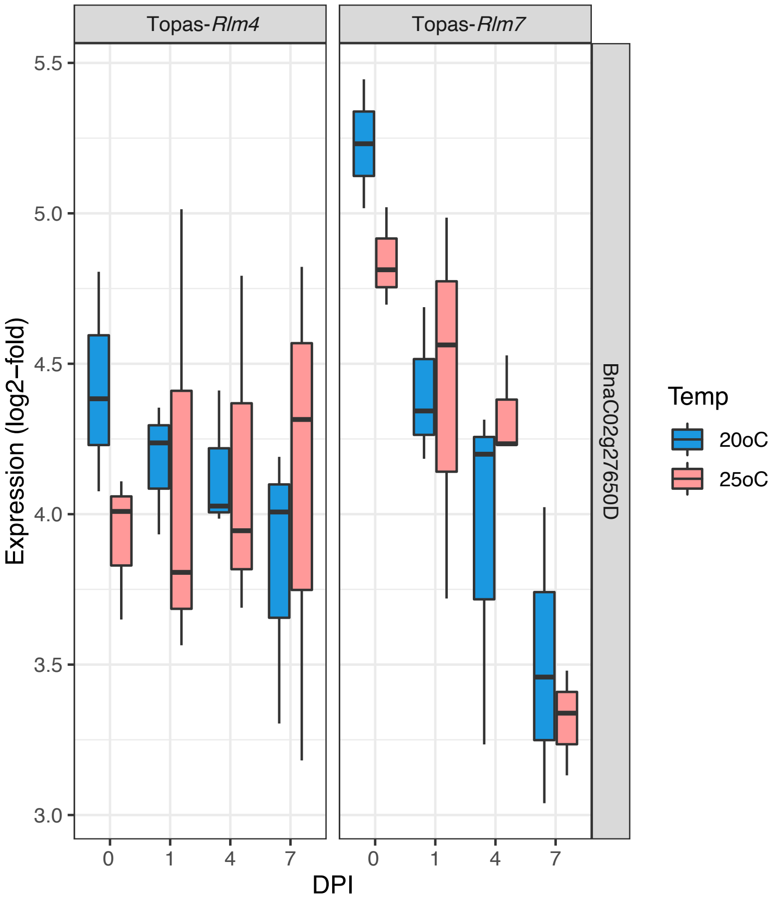

Supplementary Fig. S11 | Expression of *WRKY22* after inoculation of the *Brassica napus* introgression lines Topas-*Rlm4* and Topas-*Rlm7* with *Leptosphaeria maculans*. Expression is based on normalised log_2-transformed RNA sequencing data using DESeq2. DPI refers to days post-inoculation.

Supplementary Table 3 | Test for the presence of positive selection at the WAKL10 locus of chromosome A07 in *Brassica napus*.

Gene name	Gene ID	Locus	Sequences	[a]Model	[b]d_N/d_S	[c]lnL	[d]χ^2	*P* value	[e]Positively selected sites
WAKL10	BnaA07g20220D, BnaC06g19670D	*Rlm4/7/9*, homeolog	7	Nearly neutral	0.481	-8585.7	25.6	0.000003	
				Positive selection	0.577	-8572.9			**Ile78**, His272, **Leu275**
				[f]beta	0.438	-8584.7	31.2	<0.000001	
				[f]beta & ω	0.521	-8569.1			**Ile78**, His272, Ser274, Leu275, Leu403

[a] Nearly neutral model parameters: $\omega_0<1, \omega_1=1$; positive selection model parameters: $\omega_0<1, \omega_1=1$, $w_2>1$; ω is the

[b] Average nonsynonymous to synonymous substitution rates

[c] lnL: log likelihood

[d] Likelihood ratio test: $\chi^2 = 2 \times (\ln L_{selection} - \ln L_{neutral})$, df = 2.

[e] The WAK_GUB domain extends from position 26-75, the WAK domain from position 178-285 and the protein kinase domain from position 401-675 relative to the BnaA07g20220D peptide sequence. Residues in bold have a posterior probability > 99%, others are > 95%.

[f] The beta models are more powerful but less robust than nearly neutral vs. positive selection models.

Supplementary Table 19 | Sequence motifs in the promoter regions of *WAKL10* genes on chromosomes A07 and C06 of *Brassica napus*.

Sequence Name	Name	Sequence motif[1]	Minimum	Maximum	Length	Reference
Consensus	W box	CTGACT	3,358	3,363	6	Yamamoto et al. (2004) Biochim. Biophys. Act. 1679, 279-287
Consensus	TATA box	TATTTAA	3,335	3,341	7	Zhu et al. (2002) Plant Cell 14, 79-803
Consensus	GT1 element	GAAAAT	2,960	2,967	8	Buchel et al. (1999) Plant Mol. Biol. 40, 387-396
Consensus	W box	TGACT	2,921	2,925	5	Yamamoto et al. (2004) Biochim. Biophys. Act. 1679, 279-287
BnaA07g20220D	ABRE-related sequence	CACGCGC	1,682	1,688	7	Kaplan et al. (2006) Plant Cell 18, 2733-2748
BnaA07g20220D	ABRE-related sequence	CACGCGT	1,723	1,729	7	Kaplan et al. (2006) Plant Cell 18, 2733-2749
BnaA07g20220D	Txn start (putative)	AAATG**C**TGTGTGG	2,054	2,054	1	https://www.fruitfly.org/seq_tools/promoter.html
BnaA07g20220D	Txn start (putative)	AGT**C**TTT	2,156	2,156	1	https://www.fruitfly.org/seq_tools/promoter.html
BnaN07g22100	Box L-like sequence	ACCATCC	1,918	1,924	7	Maeda et al. (2005) Plant Mol. Biol. 59, 739-752
BnaN07g22100	GT1 element	GAAAAT	1,932	1,937	6	Buchel et al. (1999) Plant Mol. Biol. 40, 387-396
BnaN07g22100	W box	GGTCAG	2,003	2,008	6	Yamamoto et al. (2004) Biochim. Biophys. Act. 1679, 279-287
BnaN07g22100	CCAAT box	CCAAT	1,756	1,760	5	Haralampidis et al. (2002) Plant Physiol. 129, 1138-1149

[1] Putative transcription start site is colour-coded in red.
