# Supplementary material for: Transcriptomics of temperature-sensitive R gene-mediated resistance identifies a WAKL10 protein interaction network
Source: Sci Rep. 2024 Feb 29;14:5023. doi: 10.1038/s41598-024-53643-7 (PMC10904819; doi:10.1038/s41598-024-53643-7)
Supplement: Supplementary file 3 — Supplementary Information 3. [file 41598_2024_53643_MOESM3_ESM.pdf]

Supplementary Table 2 | Single nucleotide polymorphisms in WAKL10 genes of *Brassica napus* .

Genotype	Exon	Position[1]	Codon	Amino acid	Change	Read counts/proportions	References
Topas DH	1	49, 50	UUU	Phe	dN[2]		
**Topas Rlm4**	1	49, 50	**CG**U	**Arg**	**dN**	1221/1225, 1218/1225	This study
Topas Rlm7	1	49, 50	UUU	Phe	dN		
Topas DH	1	60	AUA	Ile	dS		
**Topas Rlm4**	1	60	AU**C**	Ile	**dS**	1323/1331	This study
Topas Rlm7	1	60	AUA	Ile	dS		
Topas DH	1	99	UGC	Cys	dS		
**Topas Rlm4**	1	99	UG**U**	Cys	**dS**	1706/1713	This study
Topas Rlm7	1	99	UGC	Cys	dS		
Topas DH	1	111	CAC	His	dN		
**Topas Rlm4**	1	111	CA**A**	**Gln**	**dN**	1827/1832	This study
Topas Rlm7	1	111	CAC	His	dN		
Topas DH	1	117	CCG	Pro	dS		
**Topas Rlm4**	1	117	CC**A**	Pro	**dS**	1873/1879	This study
Topas Rlm7	1	117	CCG	Pro	dS		
Topas DH	1	153	AAC	Asn	dS		
**Topas Rlm4**	1	153	AA**U**	Asn	**dS**	1675/1681	This study
Topas Rlm7	1	153	AAC	Asn	dS		
Topas DH	1	178	CAU	His	dN		
Topas Rlm4	1	178	CAU	His	dN		This study
**Topas Rlm7**	1	178	**G**AU	**Asp**	**dN**	1025/1034	
Topas DH	1	190	CUU	Leu	dN		
Topas Rlm4	1	190	CUU	Leu	dN		This study
**Topas Rlm7**	1	190	**A**UU	**Ile**	**dN**	947/955	
Darmor *bzh*[3]	1	209, 210	CCC	Pro	dN		
**Topas Rlm4**	1	209, 210	C**U**C	**Leu**	**dN**	806/807	This study
Topas Rlm7	1	209, 210	CC**U**	Pro	dN	754/762	
**Topas DH**	1	460	**A**UC	**Ile**	**dN**	2/2	
Topas Rlm4	1	460	GUC	Val	dN		Becker et al., 2019
Topas Rlm7	1	460	GUC	Val	dN		
**Topas DH**	1	736	**U**AU	**Tyr**	**dN**	4/4	
Topas Rlm4	1	736	GAU	Asp	dN		Becker et al., 2019
Topas Rlm7	1	736	GAU	Asp	dN		
Topas DH	1	856	UCU	Ser	dN		
**Topas Rlm4**	1	856	**G**CU	**Ala**	**dN**	1412/1421	This study
Topas Rlm7	1	856	UCU	Ser	dN		
**Topas DH**	1	887	C**A**A	**Gln**	**dN**	3/3	
Topas Rlm4	1	887	CUA	Leu	dN		Becker et al., 2019
Topas Rlm7	1	887	CUA	Leu	dN		
**Topas DH**	3	1563	CC**G**	Pro	**dS**	5/5	
Topas Rlm4	3	1563	CCU	Pro	dS		Becker et al., 2019
Topas Rlm7	3	1563	CCU	Pro	dS		

**Topas DH**	3	1590	AA**C**	Asn	**dS**	5/5	Becker et al., 2019
Topas Rlm4	3	1590	AAU	Asn	dS		
Topas Rlm7	3	1590	AAU	Asn	dS		
**Topas DH**	3	1625	U**C**A	**Ser**	**dN**	2/2	Becker et al., 2019
Topas Rlm4	3	1625	UUA	Leu	dN		
Topas Rlm7	3	1625	UUA	Leu	dN		
Topas DH	3	1962	GUU	Val	dN		This study
Topas Rlm4	3	1962	GUU	Val	dN		
**Topas Rlm7**	3	1962	**C**UU	**Leu**	**dN**	1118/1127	

[1] Positions are based relative to the annotated coding sequence of BnaA07g20220D.
[2] Ratio of non-synonymous (dN) versus synonymous (dS) changes = 11/6 = 1.8 (dN/dS > 1).
[3] As reads to compare to Topas DH were not available, sequences were compared to the reference genome of Darmor bzh (Chalhoub et al., 2014)
